# Harnessing myeloid cells in cancer

**DOI:** 10.1186/s12943-025-02249-2

**Published:** 2025-03-06

**Authors:** Su-Yeon Park, Ekaterina Pylaeva, Vikas Bhuria, Adriana Rosa Gambardella, Giovanna Schiavoni, Dimitrios Mougiakakos, Sung-Hoon Kim, Jadwiga Jablonska

**Affiliations:** 1 Cancer Molecular Target Herbal Research Lab, College of Korean Medicine, Kyung Hee University, Seoul, 02447 Republic of Korea; 2 Department of Otorhinolaryngology, University Hospital Essen, University Duisburg-Essen, Hufelandstraße 55, Essen, 45147 Germany; 3 German Cancer Consortium (DKTK) Partner Site Düsseldorf/Essen, Essen, Germany; 4 Department of Hematology, Oncology, and Cell Therapy, Otto-Von-Guericke University, Magdeburg, Germany; 5 Department of Oncology and Molecular Medicine, Istituto Superiore Di Sanità, Rome, Italy

**Keywords:** Myeloid cells, Immunosuppression, Macrophages, Neutrophils, Eosinophils, Therapeutic target

## Abstract

Cancer-associated myeloid cells due to their plasticity play dual roles in both promoting and inhibiting tumor progression. Myeloid cells with immunosuppressive properties play a critical role in anti-cancer immune regulation. Cells of different origin, such as tumor associated macrophages (TAMs), tumor associated neutrophils (TANs), myeloid derived suppressor cells (also called MDSCs) and eosinophils are often expanded in cancer patients and significantly influence their survival, but also the outcome of anti-cancer therapies. For this reason, the variety of preclinical and clinical studies to modulate the activity of these cells have been conducted, however without successful outcome to date. In this review, pro-tumor activity of myeloid cells, myeloid cell-specific therapeutic targets, in vivo studies on myeloid cell re-polarization and the impact of myeloid cells on immunotherapies/genetic engineering are addressed. This paper also summarizes ongoing clinical trials and the concept of chimeric antigen receptor macrophage (CAR-M) therapies, and suggests future research perspectives, offering new opportunities in the development of novel clinical treatment strategies.

## Introduction

Myeloid cells are highly versatile cells of the innate immune system whose functions can vary significantly depending on the cancer type, tumor microenvironment (TME), and stage of the disease. Their activity spans from tumor-promoting to tumor-limiting properties, and their impact on cancer progression is largely context-dependent. Myeloid cells can be modulated by various environmental signals, which determine their pro- or anti-tumor behavior. Understanding how these signals influence cell activity is crucial for developing strategies to selectively target their tumor-supporting functions without impairing their tumor-limiting activities.

The pro-tumor activities of myeloid cells range from pro-angiogenic capacity to immunosuppression. Moreover, immunosuppressive activity of these cells can be direct or indirect – acting by virtue of stromal compartment cells such as cancer-associated fibroblasts, mesenchymal cells, endothelial cells and extracellular matrix (ECM) in the TME [1].

Myeloid cells comprise monocytes/macrophages, neutrophils, eosinophils, basophils and dendritic cells (DCs). Macrophages and neutrophils, as well as myeloid derived suppressor cells (monocytic MDSCs or polymorphonuclear MDSCs) can suppress the immune response and promote tumorigenic activity in the TME [2].

Due to their plasticity, myeloid cells can be polarized into either anti-tumor or pro-tumor states [3]. However, since myeloid cells residing in the tumor niche are involved in tumorigenesis, vascularization, invasion, metastasis and tumor progression [2, 4–10], targeting these myeloid cells can be a novel therapeutic approach for cancer patients [11, 12]. Also, chimeric antigen receptor (CAR) T cell therapy and immune checkpoint inhibitors targeting PD-1, PD-L1, or CTLA-4 have been adopted to re-invigorate T cell response in the TME via the depletion of tumor-associated macrophages (TAMs) and repolarization of immunosuppressive M2-like TAMs into anti-tumor M1-like TAM. However, CAR T cells are not effective in non-B cell cancers or solid tumors and are associated with several harmful adverse effects [13]. Recent advances in CAR engineering have therefore explored alternative immune cells, such as chimeric antigen receptor macrophages (CAR-M). Unlike traditional CAR T cells therapies, CAR-M can penetrate dense tumor tissues more effectively and are also capable of TME to help recruit T cells and other immune cells to the tumor site, thereby enhancing the overall response against solid tumors [14].

Tumor-associated neutrophils (TANs), similar to TAMs show strong tumor-associated heterogeneity [15] and have emerged as a target for cancer therapy in multiple cancers such as glioma [16], breast cancer [17], gastric cancer [18] and pancreatic cancer [15].

Eosinophils are found in several human cancers [19], where they may play both, pro- or anti-tumor roles, depending on the tumor type, the stage of malignancy, the surrounding microenvironment and their activation status [20]. Tumor-associated tissue eosinophilia as well as peripheral blood eosinophil counts have prognostic and predictive roles in human cancers [21, 22] and are emerging as important indicators for immunotherapy response [23].

Additionally, DCs, a heterogeneous group of antigen-presenting cells, play a pivotal role in preventing metastasis and suppressing tumor growth by restoring dysfunctional immune responses [20, 21]. However, tumor associated dendritic cells have been shown to exert immunosuppressive effects by secreting IL-10 and TGF-β1, and can promote tumor cell growth [24]. Recently DC vaccines are being investigated to counteract immunosuppression by MDSCs, TAMs and T-regulatory cells (T_regs_) [25, 26]. Nonetheless, clinical trials and translational studies with myeloid cell modulators, CAR T cells, or DC vaccines have not yet shown any significant success.

Thus, in this review, we will focus on the myeloid cell heterogeneity in cancer, their anti- and pro-tumor activity, and therapeutic approaches targeting myeloid cells in vitro and in vivo. We will also discuss the impact of myeloid cells on immunotherapies/genetic engineering and the clinical application of CAR-M. Finally, future research perspectives in therapeutic harnessing of myeloid cells in cancer will be suggested.

### Heterogeneity of myeloid cells in cancer

Myeloid cells can be divided into mono- and polynuclear populations. Mononuclear myeloid cells include monocytes, macrophages and dendritic cells [27], while polymorphonuclear myeloid cells comprise differentiated neutrophils, eosinophils, basophils and mast cells [28]. Among them monocytes as bone marrow-derived precursors circulate in the blood and then differentiate into macrophages in tissues [29]. Monocytes can also differentiate into antigen presenting DCs rather than macrophages by the stimuli of granulocyte–macrophage colony-stimulating factor (GM-CSF) and interleukin (IL)−4 [28]. Furthermore, classical CD14^+^ CD16^−^ monocytes express CCR2, CD64, CD62L, while non-classical CD14^low^ CD16^+^ monocytes lack CCR2 in human inflammatory diseases as two major monocyte subsets [30]. Interestingly, monocyte-macrophage lineages derived from multipotent hematopoietic stem cells in the bone marrow are reported to show anti-tumor effect [31], while accumulation of immature myeloid cells and the increase of granulocytic differentiation are involved in tumor progression [32]. Due to their plasticity macrophages can be polarized into two forms: classically activated M1 macrophages with pro-inflammatory and anti-tumor property, and alternatively activated M2 macrophages, commonly called TAMs, with anti-inflammatory and pro-tumor activity [33, 34]. Thus, M1 macrophages stimulated by IFNγ and lipopolysaccharide (LPS) show the expression of inflammatory cytokines such as IL-1β, IL-6, and tumor necrosis factor (TNF)-α, whereas M2 macrophages stimulated by IL-4 and IL-13 exhibit the expression of IL-10 and transforming growth factor (TGF)-β [35]. Mantovani et al. subdivided M2 phenotype into M2a, M2b, and M2c macrophages by different cytokine treatment [36]. M2a is induced by IL-4 and IL-13, M2b is induced by immune complexes and lipopolysaccharide (LPS), while M2c express IL-10^high^ and IL-12^low^ phenotype induced by the exposure to the immunosuppressive cytokine IL-10 and glucocorticoid hormones. M2a and M2b macrophages play an immunomodulatory role by promoting T-helper 2 cell activity, whereas M2c macrophages are associated with immunosuppression and tissue remodeling. Furthermore, M2d macrophages, also termed as TAMs, are activated by Toll-like receptors (TLRs) to express vascular endothelial growth factor (VEGF) and IL-10 [37]. In general, TAMs are associated with poor outcome and resistance to chemotherapy with immune checkpoint inhibitors (ICI) [38].

In addition, another activation state of monocytes can be distinguished—so called myeloid-derived suppressor cells (MDSC) in cancer. This population was initially described as a separate myeloid cell subpopulation, but recent studies demonstrate that these cells are just an activation state of classical myeloid cells, such as monocytes (M-MDSCs) or neutrophils (PMN-MDSCs). These cells are critically involved in tumor progression and metastasis by disturbing anti-cancer functions of T cells and NK cells [23] as the major component of the TME [39]. M-MDSCs are morphologically and phenotypically similar to monocytes, with markers of CD14^+^CD15^−^HLA-DR^low/−^ in humans [40, 41] and upregulation of nitric oxide (NO), IL-10, TGFβ, and PDL1 [42]. These cells can rapidly differentiate into TAMs [43, 44], though myeloid stem cells can be differentiated into MDSCs by IL-34 treatment [44, 45]. Furthermore, CD38 related to acquired resistance to PD-1/PD-L1 blockade and CD8^+^ T cell suppression [46] was highly expressed on MDSCs in primary human cultured blood mononuclear cells of colorectal cancer (CRC) patients compared to healthy donors [47]. In contrast, the early-stage MDSCs (E-MDSCs), similar to basophils lack both macrophage or granulocyte markers and have the features of rare immaturity and weak suppression in some diseases [48]. Hence, population of MDSCs have proinflammatory property to drive varying pathological signals including T cell suppression [49, 50]. Thus, these cells enhance production and mobilization of MDSC-like populations including damage-associated or pathogen-associated molecular patterns (DAMPs or PAMPs), endotoxins, metabolite, cytokines, chemokines, hormones and distal-acting inflammatory factors [51]. Also, MDSC-like populations derived from hematopoietic progenitors are re-routed along with granulomonocytic differentiation [52, 53] by induction of unfolded protein response(UPR) and ER stress [54], apoptotic-stress response through C/EBP-homologous protein (CHOP) [55], itaconate production [56] and tryptophan catabolite-mediated aryl hydrocarbon receptor (AhR) activation [57].

Similar to macrophages, neutrophils exist with a pro- or anti-tumor activity. There is still ongoing discussion whether tumor-supporting and tumor-limiting neutrophils represent a continuum of phenotypes or are distinct populations per se. Recent single-cell sequencing data suggest a gradient of polarization, where neutrophils assume different functional states based on cues from the TME. This raises another critical question: are neutrophils' tumor-promoting properties influenced mainly by the TME or are they determined during their development in the bone marrow through granulopoiesis?

The identification of neutrophil subpopulations with distinct functions has been a focal point. Investigators highlight the complexity and heterogeneity of neutrophil populations in cancer. Studies have identified multiple distinct neutrophil populations in the blood of cancer patients, with altered proportions compared to healthy individuals. While isolated with 1,077 g/l density gradient, blood neutrophils could be found in the fraction of high density (high-density neutrophils, HDNs) and, to the minor extend, in the low-density fraction together with mononuclear cells (low-density neutrophils, LDNs). HDNs are considered to be mature cells with cytotoxic abilities toward tumor cells, while LDNs can consist of both immature MDSCs and converted neutrophils, and are characterized with impaired functions and immunosuppressive properties [58]. In patients with solid tumors, the general proportion of neutrophils in peripheral blood is elevated, and the neutrophil-to-lymphocyte ratio (NLR) is known as a prognostic marker in the variety of malignancies [59]. Moreover, the fraction of LDN is also increased in patients with cancer [60].

TANs are found within the TME and can exhibit both pro-tumor and anti-tumor functions depending on the context. A simplified N1/N2 concept described two states of neutrophil polarization, with a continuum of phenotypes between anti-tumor (often called N1) and pro-tumor (often called N2) states [61]. Accumulation of N2 neutrophils in tumor tissue is associated with poor prognosis in cancer [62]. Potent factors that have been shown to modulate neutrophil tumoral bias are type I IFNs, which stimulate anti-tumor state of these cells [63], versus TGF-β [61], which stimulate pro-tumor bias.

Recent technological advances such as single cell sequencing, mass cytometry, and unbiased computational analysis of multiparameter data allowed to identify multiple neutrophil subsets within tumor, suggesting much more complex picture than a simple N1/N2 dichotomy [64]. Moreover, phenotypically and functionally distinct neutrophil subsets could also be identified in the peripheral blood of patients with solid malignancies, e.g. melanoma [65]. Neutrophil plasticity theory suggests neutrophils can adapt their phenotype and function based on signals from the tumor microenvironment, which may explain the seemingly contradictory pro- and anti-tumor effects observed. The exact relationships between these classifications and whether they represent distinct subtypes or plastic states of the same cell population remain areas of active research.

One of the most debated areas in neutrophil biology is the distinction between TANs and PMN-MDSCs. Both share a common lineage and exhibit significant phenotypic and functional overlap, leading to challenges in their identification and study. In mice, PMN-MDSCs are often defined as CD11b^+^Ly6G^+^Ly6C^low/int^ cells [66], while human PMN-MDSCs are characterized by a CD11b^+^CD33^+^HLA-DR^−^CD14^−^CD15^+^ profile [67]. Same markers exist also on classical neutrophils. The lack of universally accepted markers underscores the functional and biochemical heterogeneity of MDSC populations, with different subtypes emerging across cancer types and disease contexts [68]. By definition, PMN-MDSCs are a distinguished from TANs by their potent suppression of T cells, although it is generally accepted that TANs exhibit immunosuppressive effects as well.

Proliferation and mobilization of neutrophil subsets from hematopoietic organs, such as from bone marrow and spleen [69, 70] are induced by growing tumor (emergency granulopoiesis) and tumor-derived cytokines. Among them, G-CSF is known to be a major regulator of steady state and emergency granulopoiesis, playing also an important role in tumor-induced granulopoiesis [71]. Other important factors include GM-CSF [70], TNF-α [72], IL-1 [73], osteopontin [74], also chemokines CCL3 and CCL4 [75] and even hormones, such as α-melanocyte-stimulating hormone [76]. Attraction of mobilized neutrophils to the tumor site is realized via chemokines CXCL1, CXCL2, CXCL5, CXCL6, and CXCL8 (IL-8) through CXCR1/2 and CXCL12 (SDF-1) and its receptor CXCR4 [77]. Adhesion molecules are critical for neutrophil extravasation into tumor tissues. Selectins (P-selectin, E-selectin) mediate initial neutrophil rolling on endothelium, while integrins (β2 integrins) and their ligands (ICAM-1, VCAM-1) enable firm adhesion and transmigration [78]. Nonetheless, the major challenge in therapeutic targeting immunosuppressive neutrophils is not successful because of the lack of unique molecular markers that differentiate between pro- and anti-tumor neutrophil subpopulations, to avoid depletion of potentially beneficial immunoactive neutrophils.

Eosinophils represent only 3% of peripheral blood leukocytes in the steady state. During inflammation, eosinophils rapidly expand to migrate towards inflamed sites, infiltrate tissues and actively participate in inflammatory responses, host defense against parasites and cancer [79, 80]. Eosinophils recruitment to the tumor sites is driven by a combination of cytokines (i.e., IL-5), adhesion molecules (i.e., CD11a (LFA1), CD11b (MAC1), chemokines (CCL11, CCL24, CCL5), and VEGFs produced by tumor and immune cells as well as damage-associated molecular patterns (DAMPs) or alarmins produced by dying tumor cells, including high-mobility group box 1 protein (HMGB1), IL-1α and IL-33 that both attract eosinophils and enhance their survival and activation [22, 47, 80, 81].

Although a clear definition of eosinophil subsets has not been established, several reports have demonstrated heterogeneity of eosinophils among and within tissues. Under homeostatic conditions, eosinophils populate a wide range of tissue sites, such as gastrointestinal tract, adipose tissue, lung, liver, heart, thymus, spleen and bone marrow [82, 83]. Bulk [84] and scRNA-seq [85] analyses of mouse eosinophils from different compartments demonstrated that these cells are transcriptionally different among tissues, with the exception of blood and spleen eosinophils. Gurtner et al. identified five distinct eosinophil subsets: precursors, immature, circulating, basal (PD-L1^−^ CD80^−^) and active (PD-L1^+^ CD80^+^). They found that the PD-L1^+^ CD80^+^ “active” eosinophils are exclusively present in the gastrointestinal tract. In a model of bacterial infection and dextran sodium sulfate –induced colitis, the involvement of active PD-L1^+^ CD80^+^ eosinophils in host defense and immune response was shown to be dependent on IL-33 and IFN-γ signaling [85].

Eosinophils also show intra-tissue heterogeneity. In the mouse lung, two distinct subsets as Siglec-F^int^CD62L^+^CD101^low^ “resident eosinophils” (rEos) and Siglec-F^hi^CD62L^−^CD10^hi^ “inducible eosinophils” (iEos) were shown, that are recruited to the lung during house dust mite–induced allergic airway inflammation [45]. These subsets were also found in the pulmonary milieu of asthmatic patients [45]. In general, rEos are characterized by a ring-shaped nucleus, have a gene expression profile consistent with an immunoregulatory phenotype and are involved in homeostatic processes. In contrast, iEos are a subset of inflammatory cells recruited under certain pathological conditions. These iEos display a more activated phenotype, as evident by up-regulation of the activation markers CD101 and CD69, enhanced secretion of TNF-α, IFN-γ, and other cytokines, increased ROS generation and F-actin polymerization [86], the latter involved in cell–cell adhesion to target cells [87].

Eosinophils display plastic phenotype and functions under different activation states [88]. It has been shown that upon transition to the airways, eosinophils gradually up-regulate Siglec-F and CD11c, associated with changes in their morphology, suggesting a phenotypic switch [89]. Moreover, in vitro stimulation with type-1 cytokine IFN-γ or type-2 cytokine IL-4 polarized peritoneal eosinophils towards distinct transcriptional profiles which were termed “Type 1” and “Type 2” eosinophils, respectively [90]. Type 2 eosinophils expressed high levels of CD101, compatible with lung iEos, whereas type 1 eosinophils expressed CD80 and PD-L1, resembling active eosinophils from mice with colitis [85].

Few studies have investigated eosinophil subpopulations in the TME. Resident and recruited eosinophils, phenotypically resembling rEos and iEos, were found in breast cancer lung metastases [44]. Transcriptome and proteome analysis revealed no transcriptional or proteomic difference between the resident and the recruited eosinophils in the metastatic lung environment, indicating that the TME, rather than intrinsic differences between eosinophil subsets, was the main driver for eosinophils to acquire anti-tumorigenic activities independent of their origin (i.e., resident vs. recruited). Specifically, IFNγ and TNFα were identified as key upstream regulators that dictate the activation status of eosinophils facilitating CD4^+^ and CD8^+^ T cell infiltration and anti-tumor immunity [44]. The same group described an IFNγ-linked signature in activated eosinophils infiltrating colon carcinoma [46]. These findings underscore the plastic nature of eosinophils and the importance of the TME in shaping their phenotype to acquire anti-tumor functions.

### Anti- and pro-tumor functions of myeloid cells in cancer and its regulation

Accumulating evidence reveals that macrophages exert anti-tumor or pro-tumor functions by their heterogenous diversity in the TME [91, 92]. M1 macrophages polarized by GM-CSF, IFN-γ, TNF-α and LPS [93] are known to enhance proinflammatory and antitumor effect, leading to inflammation or cancer sites via activation of Th1 cell response [94, 95]. Interestingly, Kudo et al. [96] reported that PEG-*b*-P(l-Arg) nanoparticle engulfed by the activated macrophages suppressed the growth of C26 adenocarcinoma cells in Balb/c mice at a high concentration of NO, while it promoted angiogenesis at a low concentration, since M1 macrophages are known to overexpress inducible nitric oxide synthase (iNOS) to produce the cytotoxic nitric oxide (NO) from l-arginine as a substrate [97]. Similarly, M1-exosomes isolated from M1-macrophages promoted antitumor efficacy of paclitaxel in Balb/c mice bearing 4T1 breast cancer cells [98]. It is noteworthy to find the critical role of Ythdf2 deficiency in antitumor immunity of macrophages via CD8^+^ T cell response, since the percentage of CD11b^+^F4/80^+^iNOS antitumor macrophages was significantly incremented and the percentage of CD11b^+^F4/80^+^Arg1 protumor macrophages was decreased in B16-OVA cells in *Ythdf2*^*c*KO^ C57BL/6 mice bearing B16-OVA tumor cells compared to *Ythdf2*^f/f^ control mice [99]. Likewise, Modak et al. [99] demonstrated that CD11b^+^CD206^+^ TAMs are the dominant tumor-infiltrating myeloid cell population with antigen presenting and specific CD8^+^ T cell activation comparable to cross-presenting CLEC9A + DCs (cDC1) in B16-F10 and CT26 syngeneic tumor models.

In contrast, M2 macrophages are known to suppress T cell infiltration and cytotoxic T cell function, leading to impairment of the antitumor immune response [100]. macrophages residing in the tumor niche are critically involved in tumorigenesis, vascularization, invasion and metastasis and drug resistance in the TME [11]. Also, alternatively activated macrophage (M2) polarization exerts anti-inflammatory and pro-angiogenic property by secreting matrix metalloproteinases (MMP2, MMP9) and growth factors (VEGF, PDGF) which promote tumor growth, metastasis, and invasion [101]. Thus, M2-polarized macrophages are closely associated with immune suppressor cells including MDSCs, T_regs_ [102], regulatory B cells and alternatively activated neutrophils [3]. T_regs_ selectively modulate survival rate of M2-like TAMs, metabolic stability and mitochondrial integrity in the TME [103]. In addition, M2 tumor-associated macrophages are critically involved in invasion and metastasis of cancer cells through ECM remodeling [104]. Previous evidence reveals that circulating monocytes are chemoattracted to tumor cells and differentiated into TAMs by several stimuli including IL-6, IL-10, VEGF, macrophage colony stimulating factor (M-CSF, also known as CSF-1), TGF-β, GM-CSF, and chemokines [105]. Also, recruited macrophages derived from circulating monocytes are widely considered to be tumor suppressor cells, while tissue-resident macrophages are regarded as a target of cancer therapy [106–108]. Thus, TAMs can disturb anti-tumor immunity by suppressing T cells, NK cells, and NKT cells or by secreting anti-inflammatory cytokines of IL-10 and TGF-β [109]. Tolerogenic signaling following the engulfment of dying tumor cells is associated with macrophage polarization toward an M2-like pro-tumor phenotype [82]. Accordingly, TAMs are critically involved in tumor progression, drug resistance and metastasis with poor prognosis in the neoplastic patients in the TME [83, 85]. Among pro-tumor TAM subsets, angiogenesis-related genes, such as SPP1 and VEGFA were identified in TAMs [35]. Also, tumorigenic effect of SPP1^+^ TAMs observed in non-small cell lung cancer (NSCLC), with macrophages expressing FOS-like antigen 2 and CCAAT/enhancer binding protein [85]. Similarly, Han et al. [100] suggested that CD163^+^ tumor-associated macrophages are closely correlated with suppressed TIM-3^+^/PD-1^+^ T cells, since depletion of CD163^+^ macrophages significantly enhanced T cell proliferation and proinflammatory cytokine production. Furthermore, Liang et al. [110] indicated that TIAM promotes osimertinib resistance, and M2-like TAM polarization in lung adenocarcinoma, while Chen et al. [111] demonstrated that Jumonji domain-containing 6 (JMJD6) induced M2 polarization via the STAT3/IL-10 signaling axis in Lewis lung carcinoma and B16F10 melanoma cells. It is also noteworthy that Tyro3, Axl and MerTK are reported as TAM receptors that skew macrophage polarization into a protumor M2-like phenotype [112], which can be molecular targets for effective cancer therapy.

Neutrophils' anti-tumor functions are diverse and involve both direct and indirect mechanisms. One of their roles includes induction of the detachment of tumor cells from the basement membrane, facilitating tumor cell clearance [113]. Neutrophils can also kill tumor cells through direct mechanisms like Fas/FasL interactions [114], the production of reactive oxygen species (ROS) and reactive nitrogen species (RNS) [115], neutrophil extracellular traps (NETs) [116], and the release of cytotoxic granules containing neutrophil elastase (NE) [117, 118] and cathepsin G [119]. Additionally, neutrophils contribute to antibody-mediated cellular cytotoxicity (ADCC) [120] or kill antibody-opsonized cancer cells by trogoptosis [121]. Beyond their cytotoxic abilities, neutrophils can activate both; innate [122] and adaptive immune responses, enhancing T cell and B cell activity either by presenting tumor antigens, in contact-dependent manner or through the secretion of pro-inflammatory factors at the tumor site [123–126] and in tumor-draining lymph nodes [127, 128].

On the other hand, neutrophils can promote tumor progression and metastasis under certain conditions. Immunosuppressive neutrophil populations contribute to an immune-tolerant and inflammatory environment that supports cancer cell invasion and viability. Via secretion of ROS and RNS, neutrophils induce tumorigenic mutations in epithelial cells [129]. They secrete factors such as IL-17a [130], TGF-β and TNF-α [131], as well as NETs [132], which facilitate the epithelial-mesenchymal transition in cancer cells. Neutrophils also play a significant role in promoting tumor cell detachment and metastasis through the secretion of MMP-9 and BV8 [8–10]. NETs, which are released by neutrophils during inflammation, have been shown to promote metastatic cell dissemination [133] and to awaken dormant cancer cells in mice, further supporting cancer recurrence [134]. Neutrophils also contribute to pre-metastatic niche formation and to promoting tumor colonization [135, 136] this determining their prognostic significance [137]. In liver metastasis models of colorectal cancer, neutrophil-specific knockout of anterior gradient-2 ameliorated metastasis [138], highlighting the role of specific neutrophil genes in tumor support.

Neutrophils also play a crucial role in tumor angiogenesis, contributing to the formation of new blood vessels that support tumor growth and metastasis. Studies have shown that the depletion of neutrophils or inhibition of their migration into tumor tissue leads to reduced vascularization, highlighting the essential role of neutrophils in promoting tumor angiogenesis [6, 7]. Neutrophils release several pro-angiogenic factors, such as VEGF [139, 140], fibroblast growth factor 2 (FGF-2) [9], oncostatin M [8], MMP-9 [141], alarmins such as Bv8 and S100A8/9 [142, 143], and NETs [144]. These factors directly stimulate angiogenesis by promoting endothelial cell proliferation, thereby supporting tumor growth [6]. Beyond their direct role in promoting angiogenesis, neutrophils can also activate pro-angiogenic functions in other immune cells, including T_regs_ [145]. Moreover, neutrophils contribute to tumor vascularization through non-angiogenic mechanisms, such as vessel co-option and vascular mimicry, where tumor cells exploit pre-existing vessels or form vessel-like structures themselves [146–148]. Neutrophils can also influence tumor growth by supporting lympangiogenesis [149], by increasing VEGF-A bioavailability via the secretion of MMP-9 and heparanase, and by secreting VEGF-D.

Tumor-supportive neutrophils suppress both innate and adaptive immunity through various mechanisms, including the secretion of ROS [150], and immunosuppressive cytokines such as IL-10 and TGF-β [151, 152], and contact-dependent suppression via PD-L1 [153]. Metabolic deprivation significantly contributes to lymphocyte suppression. In detail, depletion of L-arginine by arginase (ARG), iNOS [154] and cysteine [155] by neutrophils reduces T cell activity and proliferation. Moreover, tumor-supportive neutrophil subpopulations can recruit T_regs_ through CD40 signaling, thereby enhancing immune tolerance within the TME [156]. Neutrophils infiltrating tumors often exhibit more potent immunosuppressive functions than their peripheral counterparts, contributing to the challenge of targeting these cells therapeutically [157].

Neutrophil polarization and function are shaped by systemic and local factors, which guide their development and migration to the tumor site. Tumor-derived cytokines, such as G-CSF, and VEGF, promote the expansion and recruitment of immunosuppressive neutrophil subpopulations, while chemokines like CXCL1, CXCL2, and CXCL8 mobilize neutrophils from the bone marrow to the tumor [158], with type I IFNs serving as a negative regulator of CXCR2-mediated neutrophil migration [159]. The CXCR4/CXCL12 axis, for example, has been shown to regulate neutrophil-tumor cell interactions, promoting neutrophil infiltration into tumors and enhancing their pro-angiogenic activities [6, 160, 161]. Moreover, IL-35 has been recently identified as a factor that promotes neutrophil infiltration and their pro-angiogenic activity within the TME [162].

The pro-angiogenic potential of neutrophils is further stimulated by cytokines such as IL-6, G-CSF, and GM-CSF, which have been shown in various studies to promote neutrophil-mediated angiogenesis [8, 163–165]. Factors within the TME, such as TGF-β and S100A8/9 further influence neutrophil differentiation and function [166, 167]. To the opposite, TNF-α [168], TLR signaling and activation by bacteria [169], type 1 [63], type 2 [170] and type 3 IFNs [171] drive the polarization of neutrophils toward anti-tumor phenotype and impair their pro-angiogenic capacity [6].

**Fig. 1:**
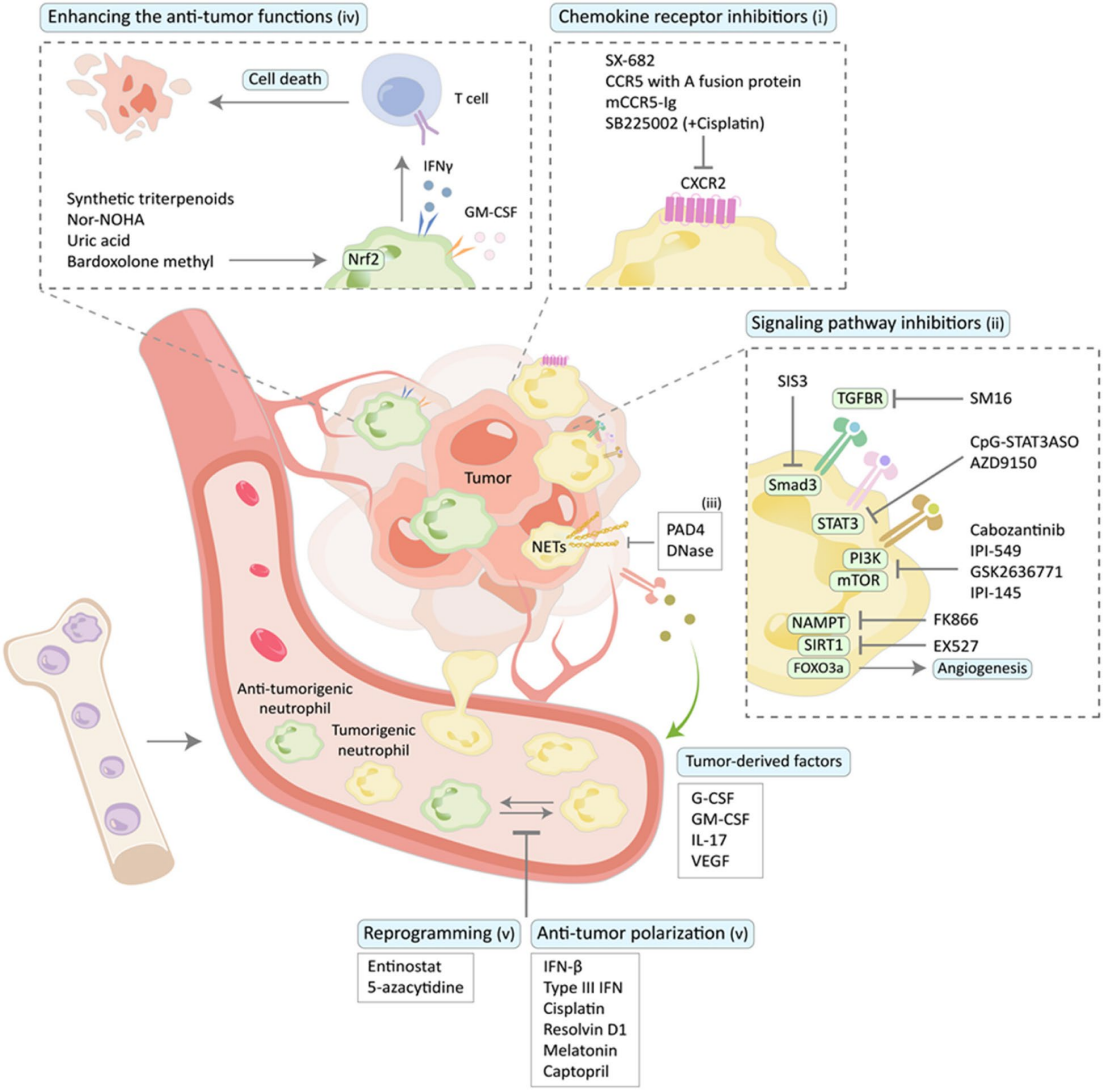
Neutrophil-targeted tumor therapy with their inhibitors and activators in cancer. (i) Inhibitors for chemokine receptor, CXCR2 promotes the expansion and recruitment of immunosuppressive neutrophils into tumor. (ii) Inhibitors of specific targets. NAMPT, SIRT1, STAT3, TGF-β receptor kinase, Smad3, PI3K/mTOR enhance tumor angiogenesis and activate the immunosuppressive property of neutrophils. (iii) Inhibition of NET formation by PAD4 or degradation of NETs by DNase can suppress tumor progression. (iv) Selective activation of Nrf2 can restore immune function and inhibit tumor progression. GM-CSF and IFNγ potentiate cytotoxic T cells response. (v) The class I histone deacetylase inhibitor entinostat and DNA demethylating epigenetic agent 5-azacytidine enhance epigenetic reprogramming of immunosuppressive neutrophils, while IFN-β, Type 3 IFN, cisplatin, resolvin D1, melatonin and captopril induce anti-tumor polarization of neutrophils by promoting anti-tumor immunity

The transcriptional regulation of neutrophil polarization is critical for their functional diversity in cancer. Immunosuppressive and pro-angiogenic potential of neutrophils is largely driven by the transcription factors STAT3 [172], Foxo3a [142], PPM1D/Wip1 [173], and NF-κB [174]. Additional transcriptional regulators, such as ERK [162] and JUNB, a member of the AP-1 transcription factor family, also contribute to neutrophils' angiogenic phenotype [10]. Frequently accepted regulators of neutrophil anti-tumor functions include STAT1 [175] and IRF8 [176], although their action is still context-dependent. For example, STAT1 is involved in the expression of immune checkpoint PD-L1 expression on neutrophils [177] and attraction of immunosuppressive neutrophils populations to the tumor site [178] (Fig. 1).

Eosinophils are a favorable prognostic indicator for colorectal cancer patients [179, 180] where their intratumoral frequency positively associates with improved survival [181, 182] and inversely correlates with malignant progression [46, 183–185]. Moreover, high levels of intratumoral eosinophils correlated with an improved survival rate in gastric cancer [186, 187] and with favorable prognosis in esophageal cancer [188, 189]. Eosinophils can have anti-tumor effects through direct or indirect mechanisms by co-operating with other immune cells in TME. In breast cancer, eosinophils infiltrating lung metastases can promote CD4^+^ and CD8^+^ T cell recruitment [44]. In mouse melanoma models, eosinophil recruitment induced by T_regs_ depletion [190] or by IL-33 treatment [191] mediated melanoma regression through production of CD8^+^ T cell attracting chemokines (i.e., CXCL9, CCL10 and CCL5) by promoting tumor-reactive CD8^+^ T cell responses. IL-33 also prevented the formation of experimental pulmonary metastasis in an eosinophil-dependent manner without an involvement of CD8^+^ T cells, indicating direct anti-tumor activity [191]. Consistently, IL-33 reduced tumor growth in colorectal cancer models through the recruitment and the activation eosinophils augmenting their cytotoxicity [192]. In colorectal cancer, tumor-infiltrating eosinophils consisted of degranulating eosinophils induced tumor rejection independently from CD8^+^ T cells [46].

Eosinophils are equipped to kill tumor cells. Their cytoplasm is characterized by the presence of granules containing cationic proteins, such as major basic proteins (MBPs), eosinophil cationic protein (ECP), eosinophil peroxidase (EPX), eosinophils-derived neurotoxin (EDN), as well as Granzymes endowed with potent cytotoxic functions [193]. The release of these secretory products through degranulation causes tumor cell death and this process can be enhanced by several soluble mediators, including IL-5, IL-33, CCL11 and IFN-γ [46, 191, 192, 194–196]. Activated eosinophils up-regulate adhesion molecules (i.e., CD11a, CD11b, ICAM-1) and Siglec-F to enhance their survival and adhesion to tumor cells, thereby leading to contact-dependent degranulation and tumor killing [194, 197].

A recently described mechanism through which eosinophils may control tumor growth is via release of extracellular vesicles (EV). IL-33 was shown to both stimulate the secretion and to qualitatively affect the molecular cargo of EV released by eosinophils. Following incorporation of EV from IL-33 activated eosinophils, melanoma cells underwent transcriptional reprogramming by increased expression of cyclin-dependent kinase genes and up-regulation of E-Cadherin, while down-modulating N-Cadherin. As a result, melanoma cells underwent cell cycle arrest, acquired a less aggressive phenotype, a reduced cell elongation and migratory extent in vitro and impaired capacity to metastasize to lungs when injected into syngeneic mice [198]. This evidence reveals an EV-mediated mechanism through which eosinophils shape tumor cell phenotype and suggest the possibility to harness eosinophil-derived EV for therapeutic purposes.

On the other hand, eosinophils constitute an unfavorable prognostic factor for overall survival In Hodgkin’ lymphoma and T cell leukemia/lymphoma [199, 200]. Moreover, high percentage of intratumoral eosinophils was associated with accelerated tumor progression and a worse overall survival in cervical carcinoma [201, 202]. Mechanistically, TSLP derived from cervical cancer cells up-regulated the levels of anti-inflammatory cytokines (IL-10, IL-4, IL-5 and IL-13) in eosinophils, which in turn promoted the proliferation and restricted the apoptosis of tumor cells [202]. Moreover, cervical cancer cell-derived TSLP increased the secretion of VEGFA and IL-8 by eosinophils, thus promoting angiogenesis in HUVEC cells [203]. Human eosinophils secrete a plethora of pro-angiogenic factors that support tumor angiogenesis in vitro [204].

Through the production of a number of soluble mediators, eosinophils may facilitate immunosuppressive responses and tumor progression. Eosinophils secrete IL-13 that orchestrates macrophage polarization towards M2 immunosuppressive phenotype [205]. Through the production of indoleamine 2,3-dioxygenase, eosinophils inhibit T cell and NK cell functions [206] fostering immune escape in human non-small cell lung cancer [207]. Under the control of IL-5 axis, eosinophils can express CCL22 that supports the recruitment of T_regs_ facilitating the colonization of lung metastasis in different tumor models [208]. Moreover, eosinophils expressing MMP-9 were found to cooperate with monocytes, neutrophils and fibrocytes to facilitate metastatic colonization of colon cancer cells to the liver [209].

### Therapeutic targets and in vivo reprograming

Recently research efforts have increased to repolarize TAMs or M2-like macrophages into M1-like macrophages for efficient target therapy [210, 211]. Generally, therapeutic strategies to harness myeloid cells include inhibition of myeloid cell production, recruitment, direct depletion, regulation of cell surface receptors and reprogramming of myeloid cells [212]. In details, M-CSF and GM-CSF as hematopoietic growth factors regulate the number or functions of monocytes or macrophages and also mediate differentiation, leading to tumor phenotype of myeloid cells in the TME [213]. Regarding myeloid cell recruitment by chemokine intervention, CXCL2 and CXCL8 chemokines produced by monocytes enhance the accumulation of neutrophils in tumors through recruitment and inhibition of apoptosis [214]. Also, CD47 and its receptor signal regulatory protein α (SIRPα) suppresses the phagocytosis of macrophage via activation of transcription factors including NFκB, Myc, and HIFα [215]. Hence, blockade of CD47/SIRPα signaling was regarded to increase phagocytosis by TAMs in solid tumors and hematological malignancies [216, 217]. Another important target is the macrophage-colony stimulating factor CSF-1 receptor (CSF-1R) related to monocyte recruitment in the TME [218, 219]. Thus, CSF-1R blockade by using monoclonal antibody (RG7155) induced cell death of CSF-1-differentiated macrophages and also reduced F4/80^+^ TAMs along with an increase of the CD8^+^/CD4^+^ T cell ratio [220]. Interestingly, Fujiwara et al. reported that PLX3397 (pexidartinib) as a potent inhibitor of CSF1R approved by the Food and Drug Administration (FDA) reduced pERK1/2 and M2 polarization induced by CSF1R and also tumor growth and lung metastasis in vivo by depletion of TAMs and FOXP3^+^ T_regs_ and enhancement of infiltration of CD8^+^ T cells into primary and metastatic osteosarcoma [43, 221]. Likewise, inactivation of phosphatidylinositol-3-kinase γ (PI3Kγ) is a potent target to reduce tumor growth and increase survival rate [130, 222]. Also, Zheng et al. demonstrated that VSIG4 induces immunosuppressive activity in M2-TAMs and T_regs_ in clear cell renal cell carcinoma (ccRCC) microenvironment [199]. Accumulating evidence reveals that TLRs as cell surface receptors recognize pathogen-associated molecular patterns (PAMPs) as innate immune receptors [200]. Among TLR family, Imiquimod as one of the TLR7 ligands induced DC activation in vitro and in vivo, leading to increase of T cell responses in skin cancers [201], which was enhanced by TAM blockade or IL-10 inhibition [202]. As an agonist of TLR7 and TLR8, resiquimod (R848)-loaded β-cyclodextrin nanoparticles (CDNP-R848) induced a repolarization of M2-like TAMs shifted toward a M1-like TAM phenotype, leading to the reduction of tumor growth in multiple murine tumor models [203]. Similarly, FimH, an adhesion portion of Escherichia coli, enhanced dendritic cell activation and also polarized M2 macrophages into M1 macrophages dependent on TLR4 and myeloid differentiation factor 2 (MD2) in B16 melanoma bearing mice [205, 206]. It is well-known that C–C motif ligand 2 (CCL2) as a monocytic chemotactic protein 1 (MCP-1), recruits macrophages or neutrophils for the immune response with its receptors such as CCR2, ACKR1, and ACKR2 on infection and tissue damage [208]. Hence, it is noteworthy that anti-CCL2 antibody or bindarit is attractive to suppress with the CCL2/CCR2 axis, since it reduced tumor multiplicity in the C3(1)/SV40Tag mouse model of breast cancer by inhibition of inflammatory mediators and macrophage markers [209].

Recent evidence reveals that exosomal microRNAs (miRNAs) derived from TAMs can modulate cancer in either an anti-tumor or pro-tumor fashion, based on the type of macrophages and the specific miRNAs [184]. Among these miRNAs, miR-9, miR-127, miR-155, and miR-125b promote M1 polarization, whereas miR-124, miR-223, miR-34a, let-7c, miR-132, miR-146a, and miR-125a-5p induce M2 polarization in macrophages [185]. Indeed, overexpression of miR155 induced repolarization of M2-activated macrophages into pro-inflammatory M1-like macrophages [186] and also reduced tumor progression of esophageal cancer via downregulation of FGF2, IL-10, Arginase-1 and IL-22 [187]. Likewise, overexpression of miR125b enhanced M1 like activation, leading to cytotoxicity against EL4 cancer cells [188]. Interestingly, miR-511-3p, encoded by MRC1 genes in mouse and humans, decreased pro-tumor activity of MCR1 in MRC^+^ TAMs and reduced the growth of murine Lewis lung carcinoma (LLC) cells [189].

Many animal studies have been conducted to find out potent agents or therapeutics to repolarize pro-tumor M2-like macrophages into anti-tumor M1 like macrophages during modern decade. Indeed, Henau et al., reported that PI3K-γ inhibitor IPI-549 significantly enhanced CD8^+^ T cell activation and reduced immunosuppression and resistance to immune checkpoint therapy with anti-CTLA-4 or anti-PD-1/anti-PD-L1 by increasing the M1/M2 ratio in 4T1 breast carcinoma and B16-GM-CSF melanoma models [190]. A lot of animals and clinical trials have been conducted with CD47 (also known as IAP) antagonist alone or combination since CD47 was identified as a “marker of self” on murine RBCs [191]. Among them, Luo et al. showed that combination therapy with an IL-15 agonist NKTR-255 and a CD47 blockade magrolimab enhanced chimeric antigen receptor (CAR)-NK cytotoxicity and macrophage phagocytic activity along with decreased primary tumor growth and prolonged animal survival in PDX-based Ewing sarcoma (ES) xenograft mouse models [192]. Previous evidence also reveals that CD47 inhibitor, namely the engineered SIRPα variant CV1 significantly in combination with IgG4 phagocytosis inducer increased the phagocytic activity of macrophages and suppressed tumor growth of xenografts in mice [193]. CSF1-R inhibition with pexidartinib reduced M2 like macrophages and enhanced repolarization into M1 macrophages with growth inhibition of follicular lymphoma in SCID mice [194]. Recently, Nguyen et al. also showed that multispecific antibody neutralizing TGF-β (CSF1R/CCR2/TGF-β) repolarized TAMs into an anti-tumor M1-like phenotype, reduced monocyte chemoattractant protein secretion in tumor tissues and spheroids [195]. Likewise, Poh et al. [196, 197] demonstrated that inhibition of hematopoietic cell kinase (HCK) reduced alternative activation of TAMs and also attenuated the growth of colon cancer xenografts along with enhancement of immunotherapy, since high HCK expression correlates with a reduced survival of colorectal cancer patients. vv. In contrast, Chen et al. [198] reported that HCK-knockout or HCK-inhibitor decreased macrophage M1-like pro-inflammatory polarization in RAW264.7 cells and bone marrow-derived macrophages. Another key regulator of M2 TAM gene expression is TMP195 for the inhibition of histone deacetylases class IIa. TMP195 treatment reduced the rate of breast cancer and pulmonary metastases by modulating macrophage phenotypes, reduced tumor burden related to pulmonary metastasis by modulating the TAM phenotype in the murine MMTV-PyMT breast tumor models along with combination therapy potential with paclitaxel [223], which was supported by findings that TMP195 increased the proportion of M1 macrophages by promoting polarization [224]. Interestingly, TLR9 agonist CpG DNA induced reprogramming of TAM from a M2-like to a M1-like phenotype, alone or in combination with an antiIL-10R Ab when injected intratumorally in 4T1 breast tumor bearing mice [225]. In addition to the repolarization of TAMs, CpG DNA stimulated a cytotoxic T cell response in the murine EG7-OVA lymphoma model [226]. Furthermore, combination therapy with TLR7 and TLR9 agonists 1V270 and SD-101(CpG) and immune checkpoint inhibitor (anti-PD1 mAbs) successfully induced tumor regression along with an increased ratio of M1-like to M2-like TAMs in murine models of head and neck squamous cell carcinoma [227]. Likewise, combination of CD40 agonist (FGK45) and gemcitabine induced tumor regression by targeting tumor-infiltrating macrophages involved in cancer inflammation in KPC mice (mutant KrasG12D and p53R172H) and human patients with pancreatic ductal carcinoma, since CD40 activation can disturb immune suppression and promote anti-tumor T cell response [228]. In combination with conventional chemotherapy (temozolomide), the immunogenic cell death (ICD) based DC vaccines enabled an increased survival and complete tumor rejection [229]. Similarly, DC-based high hydrostatic pressure (HHP) lung cancer vaccine decreased the number of CD4^+^CD25^+^Foxp3^+^ T_regs_ and Increased IFN-γ-producing tumor antigen-specific CD4^+^ and CD8^+^ T cells from non-small cell lung cancer (NSCLC) patients. Also, combination of DC-based active cellular immunotherapy (DCVAC/LuCa) and docetaxel inhibited tumor growth of lung cancer and HPV-associated TC1 tumors in mice [230].

Neutrophil modulation in cancer therapy is a rapidly evolving field, with several strategies focusing on altering the pro-tumor and anti-tumor balance of neutrophils. These approaches include targeting neutrophils' immunosuppressive properties, promoting their anti-tumor activities, administration of anti-cancer neutrophils, and using them as vectors for therapeutic delivery.

A major therapeutic strategy is depletion of tumor-supporting neutrophil subpopulations involved in tumor progression, which was explored in both preclinical and clinical studies. Type I IFNs were demonstrated to induce apoptosis of neutrophils thus contributing to the elimination of pro-tumor neutrophils [231]. Low doses of chemotherapeutic agents, such as gemcitabine and 5-fluorouracil, have been shown to reduce the viability of MDSCs, effectively diminishing their suppressive influence on the immune system [232–235]. Similarly, in human cancers, anti-CD33 therapies like AMV564, a tetravalent bispecific antibody targeting CD33/CD3, have been used to reduce MDSCs and enhance T cell function in patients with myelodysplastic syndromes [232]. Gemtuzumab ozogamicin, an anti-CD33 antibody–drug conjugate, has shown potential in targeting MDSCs in acute myeloid leukemia [236] and can potentially be extended to solid tumors. Other compounds were demonstrated the suppressive effect on MDSCs viability and accumulation in tumor tissue, including apolipoprotein E (ApoE) [237], LXRβ agonists, such as GW3965 and RGX-104 [237], a novel therapeutic peptide-Fc fusion protein that targeted the S100A family proteins [238]. Combination of neutrophil depletion with chemotherapy significantly potentiated the effect of the later [239].

Another promising approach involves improvement of neutrophil differentiation. Tumor-derived factors like G-CSF, GM-CSF, IL-17 and VEGF drive the expansion and recruitment of MDSCs. Therefore, inhibiting these growth factors or their downstream pathways may impair the formation of tumor-supportive neutrophil subpopulations [158, 240, 241]. Low doses of tyrosine kinase inhibitors deplete immunosuppressive neutrophil subpopulations via blockade of VEGF and c-KIT signaling [242]. Agents such as all-trans retinoic acid [243], paclitaxel [244] and CpG oligonucleotides [245], casein kinase inhibitor tetrabromocinnamic acid [246] have been shown to induce the differentiation of immature MDSCs into mature myeloid cells, reducing their immunosuppressive potential. Epigenetic reprogramming is a novel avenue to target the pro-tumorigenic properties of MDSCs. The class I histone deacetylase inhibitor entinostat [247, 248], DNA demethylating epigenetic agent 5-azacytidine [249] neutralized immunosuppressive neutrophil subpopulations through epigenetic reprogramming.

Other approaches involve inhibition of recruitment of tumor-supporting neutrophil subpopulations [250, 251] by inhibiting chemokine receptors. Blocking CXCR2 with inhibitors like SX-682 [238], CCR5 with A fusion protein, mCCR5-Ig [252] has been shown to reduce MDSC recruitment and enhance the efficacy of checkpoint inhibitors in preclinical cancer models. Inhibition of synthesis of neutrophil-mobilizing factors such as CXCR2 [253, 254] or GCSF [241, 255] results in the similar effect. Moreover, suppression of chemokine-independent migration by colchicine or its derivates [256] reduced infiltration of tumor tissue with neutrophils and suppressed tumor growth and metastasis.

The importance of neutrophils in the development of tumor vasculature dictates the necessity of targeting of neutrophil pro-angiogenic properties. Type I IFNs strictly control neutrophil polarization, significantly limiting their angiogenic and pro-metastatic potential [257]. In the absence of type I IFN signaling, activation of the nicotinamide phosphoribosyl transferase (NAMPT) and its downstream target SIRT1 controls the activation state of transcription factor FOXO3a, which governs the expression of pro-angiogenic genes in neutrophils. Thus therapeutic inhibition of NAMPT with FK866 [258] or SIRT1 with EX527 [142] led to significant reduction of tumor angiogenesis and suppressed tumor growth. Interestingly, a combination of neutrophil-targeting strategies with anti-angiogenic therapies has shown a promising effect in overcoming resistance to conventional anti-angiogenic treatments. For example, the use of anti-G-CSF or anti-Bv8/PROK2 antibodies has been demonstrated to enhance the efficacy of anti-VEGFR2 therapy, providing a more comprehensive approach to inhibition of tumor angiogenesis [259].

Strategies to inhibit neutrophil immunosuppressive functions focus on silencing key signaling pathways or neutralizing immunosuppressive products. STAT3 is a critical regulator of tumor-supporting neutrophil phenotype, driving their development and immunosuppressive functions. The use of antisense oligonucleotides, such as CpG-STAT3ASO or AZD9150, has been shown to effectively silence STAT3 in myeloid cells, enhancing anti-tumor immune responses [260, 261]. Interfering with TGF-β signaling on various levels, including inhibition of TGF-β receptor kinase with SM16 [61] or inhibition of Smad3 with its inhibitor SIS3 [262] promoted neutrophil anti-tumor polarization. Another approach involves targeting receptor tyrosine kinase signaling, which is critical for the immunosuppressive activity of neutrophils and other myeloid cells. Agents such as Cabozantinib [263] and dual PI3K/mTOR inhibitors [264] have been shown to inhibit this pathway, reducing the pro-tumorigenic effects of neutrophils. Similarly, isoform-selective PI3K inhibitors like PI-549 [265], GSK2636771 [266], and IPI-145 [267], among others, have demonstrated efficacy in blocking the immunosuppressive signaling of neutrophils. Furthermore, COX-2 inhibitors like celecoxib [268, 269] and phosphodiesterase-5 (PDE-5) inhibitors such as sildenafil and tadalafil [270–272] have been employed to block the upstream regulation of immunosuppressive enzymes such as arginase, which contributes to neutrophil-mediated immune suppression. Additional approaches to targeting neutrophils include the inhibition of S100A8/A9 and its receptor RAGE [167]. Selective activation of Nrf2 using synthetic triterpenoids, such as CCDO-IM and CCDO-Me [273], targeting iNOS with nitroaspirin [274], use of N-hydroxy-nor-l-arginine (nor-NOHA) [150], uric acid [275], and bardoxolone methyl [276] have been shown to inhibit the production of these immunosuppressive molecules, thereby restoring immune function and inhibiting tumor progression. These strategies aim to block the interactions and signaling cascades that contribute to neutrophil-driven immune suppression and tumor progression. Additionally, the formation of NETs, which are implicated in tumor progression and metastasis, can be targeted therapeutically. Inhibitors of protein-arginine deiminase 4 (PAD4) have been developed to prevent NET formation [277], while agents like DNase and DNase I-coated nanoparticles can degrade existing NETs [278]. Other interventions, such as the use of antibodies against NET-remodeled laminin [134] or heparin that sequesters histones from NETs [279], aim to reduce the pro-tumorigenic effects of NETs and enhance anti-tumor immune responses.

This provides the rationale for enhancing the anti-tumor functions of neutrophils as another therapeutic approach. Type I IFNs, particularly IFN-β, play a crucial role in inducing anti-tumor polarization of neutrophils, transforming them from a pro-tumor to an anti-tumor phenotype characterized by enhanced tumor cytotoxicity, elevated expression of anti-tumor markers such as MHC1, ICAM1 and TNF-α, potent capacity to stimulate cytotoxic T cells [171, 280]. Moreover, they are necessary for the β-glucan-induced trained immunity [281]. Type III IFNs through the different receptors activate the same intracellular signaling cascade and thus can be used for the induction of anti-tumor phenotype in neutrophils in the deficiency of type I IFN signaling [171]. GM-CSF and IFN-γ were demonstrated to be responsible for the antigen-presenting cell-like phenotype and strong capacity to potentiate anti-tumor cytotoxic T cells responses [124]. Therapeutic activation of neutrophils by TNF-α, CD40 agonist, and tumor-binding antibody contributes to massive ROS production and tumor eradication independently of T cells [115]. Cisplatin and other chemotherapeutic agents have been shown to polarize neutrophils into an anti-tumor phenotype, which is characterized by enhanced cytotoxicity and the promotion of T cell infiltration. In particular, cisplatin-induced ferroptosis in tumor cells has been linked to anti-tumor polarization of neutrophils, transforming the TME into a more immunogenic state, conducive to immune cell attack [282]. This strategy highlights the potential of combining classical chemotherapy with neutrophil modulation to improve treatment outcomes. Several other therapeutic approached were confirmed to induce anti-tumor polarization of neutrophils, thus facilitating anti-tumor immunity, including resolvin D1 [283], melatonin [284], captopril [285] (Fig. 1).

Neutrophils were also demonstrated to be polarized towards the antitumor phenotype by discoidal polymer micrometric ‘patches’ that adhere to the neutrophils’ surfaces without being internalized. Such micropatch-activated neutrophils effectively activated innate and adaptive immune responses in lymphatic organs, resulting in tumor regression [286].

Recent evidences added insights into the mechanisms by which eosinophils may control tumor growth in the TME and have shown strategies to harness these cells for therapeutic purposes. In a recent translational study, a higher prevalence of activated eosinophils was found in the tumor tissue of esophageal cancer patients at early stages with respect to late stages [287]. These eosinophils displayed a transcriptomic profile unveiling a role in oxidative stress and tumoricidal activities in vitro. Of interest, treatment with intranasal rIL-5 attenuated the number of tumors in a mouse model of inducible esophageal carcinoma, providing the first preclinical evidence of a therapeutic benefit of eosinophil expansion in tumor growth control. Moreover, it was reported that estrogens negatively regulate the number of peripheral and intratumoral eosinophils, and this contributes to increased tumor growth in breast cancer and melanoma models [288]. Inhibition of estrogen receptor signaling decreased tumor growth and increased the efficacy of immune checkpoint inhibitors (ICI) in an eosinophil-dependent manner. Another targetable negative regulator of eosinophil accumulation recently described is the secreted enzyme autotaxin (ATX). ATX suppressed CCL11-driven infiltration of eosinophils into the pancreatic tumor microenvironment to facilitate tumor progression. Genetic or pharmacologic ATX inhibition increased the number of intratumor eosinophils, which promote tumor cell death locally and suppress tumor progression [289].

Eosinophils exhibit a prognostic/predictive role in cancer immunotherapy [23]. In metastatic melanoma patients, high absolute eosinophil count (AEC) in the peripheral blood at baseline or an increase of AEC following immunotherapy is a predictor of response to ICI and correlates with prolonged survival [4, 290–294]. Moreover, a significant association between high AEC and better clinical outcomes has been reported in non-small lung cancer patients treated with ICI [295–297].

Eosinophils play an active role in the anti-tumor response following ICI therapy. Degranulating eosinophils that infiltrate melanoma were identified within tumors following ICI treatments and positively correlated with CD8^+^ T cell numbers and the effectiveness of immunotherapy [293]. In breast cancer models, eosinophils mediated the anti-tumor effects of CTLA4 blockade through vascular remodeling. Administration of anti-CTLA4 promoted eosinophil intratumoral accumulation via stimulation of T cell production of IL-5, CCL5 and CCL11. Eosinophils were an important source of IFN-γ which mediated vascular normalization. In vivo depletion of eosinophils abrogated the effect of anti-CTLA4 therapy on tumor vessel normalization and dampened its therapeutic efficacy [298]. In a translational study, circulating eosinophils increased in metastatic triple negative breast cancer patients who responded to ICI. By using spontaneous mouse models of primary and metastatic breast cancer the authors demonstrated that anti-PD1 plus anti-CTLA4 immunotherapy increased IL-5 production by CD4^+^ T cells, which stimulated eosinophilopoiesis from the bone marrow and systemic eosinophil expansion, but not intratumoral infiltration. The combination of ICB plus cisplatin was able to induce IL-33 intratumoral expression which was responsible for infiltration of eosinophils and eosinophil-dependent CD8^+^ T cell responses. Of note, RNA-seq identified enrichment for IFN-γ signature in tumor-infiltrating eosinophils upon ICI-cisplatin therapy [299]. In models of hepatocellular carcinoma and breast cancer, administration of an inhibitor of dipeptidyl peptidase DPP4, a type II transmembrane protein that negatively regulates lymphocyte trafficking, increased the levels of CCL11 promoting the migration of eosinophils into tumors. Tumor control was abolished after depletion of eosinophils or treatment with degranulation inhibitors. Furthermore, tumor-cell expression of IL-33 was necessary and sufficient for eosinophil-mediated anti-tumor responses and this mechanism contributed to the efficacy of ICI therapy [300].

Overall, these studies not only unveil the critical role of eosinophils during ICI-mediated immune response and therapeutic success but also highlight the importance of activating stimuli, such as IFN-γ and IL-33, for tumor recruitment and induced anti-tumor responses of eosinophils.

### Clinical trials

The variety of clinical trials have been conducted with macrophage modulators recently (summarized in Table 1). Among them, clinical trials with monoclonal anti-human CD47 antibody (Hu5F9-G4, CC-90002, SRF231, IBI188) or recombinant SIRPα-Fc protein (TTI-621, ALX148, TTI-622) were applied as a monotherapy or combination with immune checkpoint inhibitors in patients with solid tumors or hematologic malignancies in clinical trial Phase I (NCT02216409, NCT02953782, NCT03558139, NCT02367196, NCT02663518, NCT03013218, NCT03512340, NCT03530683, NCT03717103. In particular, the clinical study (NCT02641002) with CC-90002 reveals that all patients were dropped for clinical study due to death or adverse events of diarrhea (46.4%), thrombocytopenia (39.3%), febrile neutropenia (35.7%), and aspartate aminotransferase (35.7%) [301]. In addition, more than 30 clinical trials were conducted in several cancers with colony-stimulating factor-1 receptor (CSF1R) inhibitors or CCR2 inhibitors: AMG 820 [302], LY3022855 ((NCT02265536; Immune PD reported, no efficacy) [303], emactuzumab (RG7155), cabiralizumab [304], lacnotuzumab (MCS110) [305], ARRY-382 ((NCT02880371; combination tolerated but limited efficacy) [305], BLZ945 [306], pexidartinib [307] and edicotinib [308]. Among CSF1R inhibitors, though pexidartinib and edicotinib showed some clinical efficacy in tenosynovial giant cell tumors [307, 308], while most trials with CSF1R or CCR2 inhibitors were initiated with lacking preclinical evidence and modest clinical efficacy by reducing the number of circulating monocyte, TAMs and increasing the number of tumor T cell [309, 310]. In contrast, combination of PF‐04136309 (PF-6309; CCR2 inhibitor) and FOLFIRINOX (leucovorin, fluorouracil, irinotecan and oxaliplatin) chemotherapy weakly reduced circulating monocytes and increased the number of T cells in patients with pancreatic carcinoma in a clinical trial (NCT01413022) [311] but is was discontinued because of safety concerns (NCT02732938). Interestingly, Casanova-Acebes et al. [312] suggested that CCR2 inhibitors might not be effective in non-small cell lung cancers due to the compensation by TAMs or M-MDSCs lacking CCR2. Also, HuMax-IL8 (now known as BMS-986253) treatment weas safe and well tolerated in 15 patients with metastatic or unresectable locally advanced solid tumors by a clinical study (NCT02536469) [313] whereas CCR2/5 antagonist BMS-813160 showed excellent oral bioavailability and inhibited the migration of inflammatory monocytes and macrophages as a clinical candidate [314]. Interestingly, PI3Kγ activated by G protein-coupled receptors plays a critical role in macrophage differentiation and neutrophil activation [315, 316]. Recent clinical trial (NCT02637531) reveals the safety and suitable doses (30 and 40 mg once daily) of a PI3Kγ inhibitor eganelisib during a Phase 2 study [317]. Also, another Phase 2 clinical trial (NCT03961698) shows the efficacy of TAM-reprogramming immunotherapy with eganelisib and its potential in combination therapy with anti-PD-L1 atezolizumab and nab-paclitaxel in the patients with triple-negative breast cancer or renal cell carcinoma [318].

**Table 1:** Clinical trials targeting myeloid cells

No of clinical trial	Cancer Type	Agents and treatment method	Targeted cells	Phase type and main results
**Regulation of neutrophil migration**				
NCT02637531	Solid tumors	Selective **inhibition of phosphoinositide-3-kinase (PI3K)-gamma** (IPI-549, eganelisib) monotherapy	TAM	Phase I/b
NCT03961698	Triple negative breast cancer, RCC	Selective **inhibition of phosphoinositide-3-kinase (PI3K)-gamma** (IPI-549, eganelisib) in combination with front-line treatment	Tum, TAM, Gra	Phase II, active not-recruiting. Local and systemic immune activation [319]
NCT01861054	Metastatic breast cancer	Reparaxin (oral** CXCR1** inhibitor) in combination with paclitaxel	Myel, Neu, Tum	Phase II, The primary endpoint of prolonged PFS was not met [320, 321]
NCT02001974	HER2-negative metastatic breast cancer	Reparaxin (oral **CXCR1** inhibitor) in combination with paclitaxel	Myel, Neu	Phase Ib, safe and tolerable [322]
NCT02370238	Metastatic triple-negative breast cancer	Reparaxin (oral **CXCR1** inhibitor) in combination with paclitaxel	Myel, Neu	Phase II
NCT03161431	Metastatic melanoma	SX-682 (**CXCR1/2**) inhibitor	Myel, Neu	Recruiting
NCT03473925	Advanced or metastatic castration-resistant prostate cancer, microsatellite-stable CRC, NSCLC	**CXCR2 (**SX-682, navarixin) oral inhibitor in combination with pembrolizumab	Myel, Neu	Phase II, Navarixin plus pembrolizumab did not demonstrate sufficient efficacy; safety and tolerability of the combination were manageable [323]
NCT02536469	Metastatic or unresectable solid tumors	HuMax-IL8 (BMS-986253) human IgG1 kappa monoclonal antibody that binds to free **IL-8**	Myel, Neu	Phase I, safe and well-tolerated; no objective tumor responses were observed, 73% had stable disease with median treatment duration of 24 weeks; reduction of serum IL-8 levels [324]
NCT03400332	Metastatic or advanced solid tumors	HuMax-IL8 (BMS-986253) in combination with nivolumab	Myel, Neu	Unknown
NCT04521764	Metastatic breast cancer	Modified measles virus, MV-s-NAP, expressing **helicobacter pylori neutrophil activating protein**, recruiting neutrophils into inflamed gastric mucosa	Neu	Phase I, pre-clinical safety assessment [325]
**Depletion or reprograming immunosuppressive neutrophil subpopulations (MDSCs)**				
EudraCT 2010–018841-76	Cervical cancer	**Myeloid cell depletion** (carboplatin-paclitaxel) prior to vaccination (HPV16-SLPs)	Myel	Fosters robust T cell responses [326]
NCT00617409	Extensive stage SCLC	**All-trans-retinoic acid (ATRA)** in combination with vaccination with dendritic cells transduced with wild-type p53	MDSCs	Depletion of MDSCs by ATRA improved the immune response to vaccination [327]
NCT02076451	Advanced cancers	**TRAIL-R2 agonistic antibody** (DS-8273a)	MDSCs	Phase I, Elimination of different populations of MDSCs without affecting mature myeloid or lymphoid cells, and tumor infiltrating cells, no objective clinical responses (CR and PR) [328]
NCT04387682	OSCC	Particulate **β-glucan** as a food-grade supplement	MDSCs	Interventional, unknown status. Promotes the human immune system via subversion of immune modulatory MDSCs
N/A	NSCLC	Yeast-derived whole **β-glucan** particles (WGP)	MDSCs	Decreased frequency of nMDSCs [329]
NCT03302247	Metastatic NSCLC	**Gemcitabine** in combination with Nivolumab	MDSCs	Reduces immunosuppression by killing myeloid derived suppressor cells, thereby increasing the efficacy of Nivolumab
NCT02259231	Unresectable or metastatic melanoma	**Omaveloxolone (RTA 408)**	MDSCs	Phase Ib, II, study, completed
NCT02868255	HCC and ovarian cancer	**Anti-Signal Regulatory Protein α (SIRPα)** antibodies (in vitro on patients-derived explants)	MDSCs	Observatioal, completed
NCT02124850	HNSCC	Motolimod (**TLR8 stimulation**) and cetuximab	MDSCs	Phase Ib, decreased MDSCs, improved T cell immunity [330]
NCT01374217, NCT00843635, NCT00752115	Various	**Phosphodiesterase-5 inhibitors**: tadalafil, sildenafil, nitro-aspirin	MDSCs	Unknown
NCT00843635	HNSCC	**Phosphodiesterase-5 inhibition** with tadalafil	MDSCs	Immunostimulation [272]
NCT00779168	Prostate cancer	**White button mushroom** (WBM) powder	MDSCs	Phase I, impact prostate cancer antigen levels and decrease immunosuppression [331]
NCT05615883	Melanoma	**ketogenic milieu induced by acute exercise** as well as the effects of recurrent exercise bouts	MDSCs	Interventional, recruiting
NCT06059651	-	Metabolic & Bariatric Surgery	MDSCs	Observational, recruiting
N/A	CRC	L-arg supplementation	MDSCs	Did not decrease tissue immunosuppression [332]
**Inhibition of tumor-supporting functions of neutrophils**				
NCT04262388	Locally advanced or recurrent/metastatic PDAC, NSCLC, and HNSCC	Durvalumab (MEDI4736) and Oleclumab (MEDI9447) (**anti-CD73**)	CD73^+^, myel	Phase II, withdrawn due to minimal clinical activity of the combination
N/A	Resectable NSCLC	Oleclumab (anti-CD73) or danvatirsen (**anti-STAT3** antisense oligonucleotide) in combination with ICI durvalumab (anti-PD-L1)	Myel	Oleclumab improved MPR rates [333]
N/A	Advanced solid tumors and lymphomas	**ROS inhibitor** Bardoxolone methyl (RTA 402; CDDO-Me)	Neu	Phase I, partial clinical response [334]
NCT02716012	HCC	**MTL-CEBPA (**small activating RNA**)** in combination with sorafenib	MDSCs, TAM	Phase I, acceptable safety profile and potential synergistic efficacy with TKIs, inactivation of immune-suppressive myeloid cells with potent antitumor responses [335]
NCT04105335	Advanced solid tumors	**MTL-CEBPA** (small activating RNA) in combination with pembrolizumab	Tum, MDSCs, Gra	Phase I, Ib interventional, active not recruiting
N/A	NSCLC, lymphoma	AZD9150 (antisense nucleotide inhibitor of **STAT3**), single-agent	Tum, MDSCs, Gra	Phase I, clinically active [336]
N/A	Metastatic cancer	Multitargeted** tyrosine kinase inhibitor** (VEGFR1, VEGFR2, VEGFR3, PDGFR, c-kit, FLT3, and RET) Sunitinib	Tum, MDSCs, T	Increased efficiency of radiotherapy by reversing MDSC and T_regs_-mediated immune suppression [337]
NCT01118351	Bladder cancer	Multitargeted **tyrosine kinase inhibitor** (VEGFR1, VEGFR2, VEGFR3, PDGFR, c-kit, FLT3, and RET) Sunitinib	Tum, MDSCs, T	Phase II, safe but not associated with improved clinical outcomes [338]
NCT03394144	Advanced solid malignancies	Antisense oligonucleotide targeting **STAT3** (danvatirsen) as monotherapy and in combination with with ICI durvalumab	Various, Neu	Phase I, Well tolerated, STAT3 expression tended to decrease [339]
NCT02390843	Refractory pediatric solid and CNS tumors	Effect of **simvastatin** on IL6/STAT3 pathway changes	Various, Gra	Phase I
NCT01373164	Unresectable pancreatic cancer	Small molecule **TGF-β receptor inhibitors** (galunisertib) in combination with gemcitabine	Various, Gra	Phase I, II, Improvement of overall survival, and 4 proteins (IP-10, FSH, MIP-1α, PAI-1) were potentially predictive for this combination treatment [340]
NCT02304419	Advanced, refractory solid tumors and tumor progression or treatment intolerance	**TGF-β inhibitor** (LY2157299 Monohydrate Galunisertib)	Various, Gra	Phase I
NCT02734160	Metastatic pancreatic cancer	Small molecule **TGF-β receptor inhibitors** (galunisertib) in combination with ICI durvalumab	Various, Gra	Phase Ib, well tolerable, Limited clinical activity, no association between potential biomarkers and treatment outcomes [341]
NCT04868747	Non-metastatic gastric cancer	Perioperative **lidocaine** on NETs	Neu	Withdrawn due to financial problems
NCT02839668	Primary breast tumor resection	I.V. perioperative **lidocaine** decreased postoperative expression of NETosis and MMP3	Neu	Decreased postoperative expression of NETosis and matrix metalloproteinase 3 [342]
NCT04840511	Prostate cancer	Perioperative **lidocaine** infusion on neutrophil extracellular trapping in the patients undergoing the robot-assisted prostatectomy	Neu	Interventional, active, not recruiting
ChiCTR2300068563	Various	**Lidocaine**	Neu	In comparison to other anesthesia modalities does not increase NETs and angiogenic markers [343]
**Stimulation of anti-tumor properties of neutrophils**				
NCT02451488; also NCT05717140	Metastatic melanoma	Neo-adjuvant perioperatively subcutaneous **GM-CSF** restores the host regional lymph node immunity	APC, myel, T	
NCT01598129	Solid tumors	ONCOS-102, an Engineered Oncolytic Adenovirus Expressing **GMCSF**	APC, myel, T	Phase I, Induced antitumor immunity
NCT03003676	Melanoma	ONCOS-102, an Engineered Oncolytic Adenovirus Expressing **GMCSF** in combination with ICI (pembrolizumab)	APC, myel, T	
NCT02429440	Advanced RCC	Synthetic adjuvant **peptide immunization patient-specific in combination with immune adjuvants** (granulocyte macrophage colony stimulating factor; Montanide ISA-51)	APC, myel, T	Phase I, II, well tolerated, immunogenic, improved survival [344]
NCT01433172	Stage IV lung adenocarcinoma	**GM-CSF-Producing and CD40L-Expressing Bystander Cell Line** (GM.CD40L) Vaccine in combination with CCL21	APC, myel, T	Phase I, II, completed well tolerated, no significant associations between vaccine immunogenicity and outcomes [345]
NCT04517539	Metastatic thymic epithelial tumors	Stereotactic body radiotherapy (SBRT) combined with **rhGM-CSF and Peginterferon α2b**	APC, myel, T	Phase II, interventional
NCT01176552	High-risk RCC	**GM-CSF, IFN-α and IL-2** postoperatively	APC, myel, T	Phase II, completed
NCT00607048	Advanced solid tumors	anti-**CD40** antibody (CP-870893) combined with carboplatin and paclitaxel	Myel	Phase I, upregulation of immune co-stimulatory molecules on T cells [346]
NCT02216409	Solid tumors	Hu5F9-G4 humanized IgG4 antibody that targets **CD47** to enable phagocytosis	Myel	Phase I, well tolerated [347]
NCT03717103	Advanced solid tumors and lymphomas	IBI188 **CD47-SIRPα** inhibitor	Myel	Phase I
NCT00911560	Neuroblastoma	Effect of oral **β-glucan** on response to ganglioside vaccine	Myel	Phase I, II increased immune response without additional toxicity [348]
NCT04710290	Metastatic cancer	**β-glucan**, glutamine, and colostrum immunoglobulin powder with β-glucan	Myel	Phase II, III, unknown status
NCT00169104	Metastatic breast cancer on chemotherapy	Effect of **G-CSF** on antibody-dependent cell mediated cytotoxicity (ADCC)	Neu, NK	Phase II, III, terminated due to achievement of primary study endpoint
N/A	c-erbB-2-overexpressing tumors	2B1 bispecific murine monoclonal antibody (BsMAb) with specificity for the c-erbB-2 and Fc gamma RIII extracellular domains-	NK, Neu, Mon	Phase I. Promotes the targeted lysis of malignant cells overexpressing the c-erbB-2 by CD16 expressing cells, safe, potent immunological consequences, minor clinical responses [349]
N/A	Stage IV, HER-2/neu positive cancer that could not be treated effectively by standard therapies	MDX-H210 (BsAb targeting Fc gamma RI and the HER-2/neu) in combination with IFNγ	Myel, NK	Phase I, enhanced phagocytosis and ADCC [350]
N/A	Breast cancer	MDX-210 alone vs a combination of G-CSF and MDX-210	Neu	Phase I, G-CSF elevated ADCC in neutrophils [351]
CO08604	Advanced melanoma	Intratumoral injection of **α-gal glycolipids** to enhance tumor antigen presentation	APC, myel	Phase I, well tolerated, tumor necrosis in biopsies [352]
NCT05056857	Breast cancer	**Tamoxifen**	Neu (NETs)	Observational, recruiting
NCT06509880	Metastatic CRC at stages T3-4 N1-2 M1	Sodium nucleinate mitochondrial immunotherapy (stimulation of mitochondrial activity)	Neu	Active, not recruiting
NCT06209229	Solid tumors	Calcitreol capsules, Magnesium B-6 capsules, products containing quercetin flavonoids, Naderin (sodium deoxeribonucleate) daily for 21 days (stimulation of phagocytosis function, liposomal activity, mitochondrial function)	Neu	Unknown status
NCT01261962	CRC	Oral **zinc supplementation** with adjuvant chemotherapy	Neu	Unknown status
NCT01831310	CRC	Postoperative **glutamine-dipeptide and/or omega 3 fatty acid** supplemented parenteral nutrition	Neu	Phase IV interventional, completed
NCT04352335	Advanced lung cancer receiving ICIs	**Immunomodulatory drugs** (Astragalus Polysaccharide) in combination with ICI	Various	Observational, completed
NCT02753127	Metastatic CRC	Napabucasin—orally administered **reactive oxygen species** generator	Various	Did not improve overall survival [353]
**Cellular therapy**				
NCT00900497	Metastatic, non-hematological cancer	**White blood cells** from healthy donors	Gra	Phase I, II, enrolling by invitation
NCT04124666	Advanced cancer	**Granulocyte** infusions from healthy unrelated donors	Gra	Phase I, II interventional, unknown status
NCT06240767	Advanced cancer with failed chemotherapy or ineffective standard treatment or relief measures	Single infusion of human **granulocytes with anti-cancer mouse characteristics** was performed for 5 consecutive transfusions at the interval of 2 ± 1 day	Gra	Phase I interventional Recruiting
NCT06496724	Recurrent/metastatic breast cancer	**Albumin-bound Paclitaxel/Granulocyte**-based therapy (intravenous injection of modified autologous granulocytes)	Gra	Recruiting
**Targeting side effects**				
various	various	**G-CSF** for prevention and treatment neutropenia	Neu	
NCT01170845	Esophageal cancer	Effect of **Neutrophil Elastase Inhibitor** on Lung Injury After Esophagectomy	Neu	Postoperative acute lung injury was significantly lower [354]
NCT03986086	Hematological	MPH966, a **neutrophil elastase inhibitor in** prevention of graft-versus-host disease after hematopoietic stem cell transplant	Neu	Withdrawn (lack of funding)
NCT00488904	Colorectal cancer	**Omega-3 fatty acids** (n-3 FA)	Neu	Anti-inflammatory effects in surgical patients, without reducing the risk of postoperative complications [355]
**CAR macrophage-based therapies**				
NCT05138458	T cell lymphoma	MT-101 (mRNA engineered CAR monocyte (CAR-M)	CD5^+^	Phase I, II, Suspended (Temporary operational pause; no safety concerns)
NCT06224738	HER2-positive Advanced Gastric Cancer with Peritoneal metastases	anti-Human epidermal growth factor receptor 2 (HER2) CAR-M	HER2^+^	Phase I ( Primary completion date: 2025–03-01)
NCT04405778	GPC3 + expressing previously treated solid tumors	TAK-102	GPC3 +	Phase I ( Primary completion date: 2026–07-04)
NCT04660929	HER2 + recurrent or metastatic solid tumors	Anti-HER2 CAR	HER2 +	Phase I (2024–12–31)
NCT06254807	HER2 + advanced (unresectable) or metastatic solid tumors	Anti-HER2 CAR-monocytes (CT-0525)	HER2 +	Phase I (2025–03–31)

*APC* antigen-presenting cells, *CAR* chimeric antigen receptor, *Gra* granulocytes, *ICI* immune checkpoint inhibitor, *MDSCs* myeloid-derived suppressor cells, *Myel* myeloid cells, *Neu* neutrophils, *NETs* neutrophil extracellular traps, *NK* natural killer cells, *T* T cells, *T*_regs_ regulatory T cells, *TAM* tumor-associated macrophages, *Tum* tumor cells, *CNS *central nervous system, *CRPC* castration-resistant prostate cancer, *CRC* colorectal cancer, *HCC* hepatocellular carcinoma, *HNSCC* head and neck squamous cell carcinoma, *NSCLC* non-small-cell lung cancer, *OSCC* oral squamous carcinoma, *PDAC* pancreatic ductal adenocarcinoma, *RCC* renal cell cancer, *SCLC* small cell lung cancer

Various neutrophil-targeting treatment options have been explored through the variety of clinical trials (summarized in Table 1). These include efforts to regulate neutrophil migration to tumor tissue, deplete or reprogram immunosuppressive neutrophil subpopulations (such as MDSCs), inhibit tumor-supporting functions of neutrophils, stimulate their anti-tumor properties, and even employ cellular therapies. Additionally, some studies have aimed at reducing the adverse effects of primary cancer treatments by targeting neutrophils. Although a few studies have reached Phase 2 trials to evaluate the impact on patient prognosis, most significant findings have been observed in earlier phases, particularly in observational or Phase 1 trials.

Among the therapeutic strategies, regulating neutrophil migration by inhibiting the CXCL-CXCR1/2 axis showed limited improvement in clinical outcomes, although it modulated inflammatory profiles [320, 321, 323, 324]. Despite the emphasis on inhibiting neutrophil migration to tumors, one study suggested that increased neutrophil infiltration in metastatic breast cancer might have beneficial effects [325].

The depletion of immunosuppressive neutrophil subsets, particularly MDSCs, yielded clear biological effects, such as immune activation, in most studies [272, 326–331]. However, data on the impact of this strategy on disease outcomes remain limited. For instance, while improved prostate-specific antigen levels were observed in prostate cancer [331], no significant effect was seen in advanced cancers [328].

Efforts to reverse the immunosuppressive properties of neutrophils have also been promising. In addition to improvements of immunological parameters, these strategies have demonstrated more consistent clinical effects. For example, inhibition of ROS [334] or STAT3 [336], and the use of multitargeted tyrosine kinase inhibitor sunitinib along with radiotherapy [337] have been effective. However, certain approaches, such as the combination of TGF-β inhibitors with immune checkpoint inhibitors (ICI) [341] or sunitinib monotherapy, failed to show clinical improvement.

Therapeutic strategies aimed at enhancing anti-tumor immune responses, such as the induction of APC, trained immunity through beta-glucan, and ADCC stimulation, have demonstrated laboratory success. However, their effect on patient outcomes remains variable and often controversial [344, 345, 349, 353].

Interestingly, some contradictory approaches have been investigated. For instance, blocking G-CSF signaling [336] was explored along with an option to use G-CSF to enhance ADCC (NCT00169104). Similarly, studies aimed at both inhibiting neutrophil extracellular traps (NETs) to mitigate negative effects [354] and stimulating NET release to increase cytotoxicity (NCT05056857) have been conducted. The overall benefit of decreasing ROS production appears to outweigh strategies that increase ROS. The question of G-CSF addition, particularly its role in preventing chemotherapy-induced neutropenia, remains open, given the substantial evidence suggesting the negative impact of STAT3 signaling on disease outcomes [356].

While some trials have aimed at selectively targeting neutrophils, the majority of clinical trials have observed neutrophil reactivation as a potential bystander effect of the therapy, which could also contribute to favorable disease outcomes. However, cellular therapy remains the most targeted option for neutrophil modulation, but most clinical trials in this area are in early stages, and data on patient outcomes are not yet available.

A few therapeutic strategies have focused on mitigating neutrophil-dependent side effects of cancer treatments, such as excessive inflammation and local complications. For instance, neutrophil elastase inhibitors have shown promising efficacy in preventing lung injury [354].

### Impact of myeloid cells on T cell based immunotherapies

T cell-based therapies have revolutionized cancer treatment by harnessing the body's own immune system to target and destroy cancer cells. Among them ICIs and CAR T cell therapies are most attractive [357]. ICIs, such as PD-1 and CTLA-4 blockers, work by removing the "brakes" on immune cells, allowing T cells to more effectively recognize and attack tumor cells. This approach has shown remarkable success in treating several cancers, including melanoma and lung cancer. CAR T cell therapy, another breakthrough, involves engineering T cells to express chimeric antigen receptors (CARs) that can specifically recognize and eliminate cancer cells. This therapy has shown significant success, particularly in treating blood cancers such as leukemia and lymphoma [358]. These innovative therapies represent a shift from conventional cancer treatments and offer a new hope for patients with intractable cancers.

Since myeloid cells, as detailed in this review and elsewhere, can significantly influence both intrinsic innate and adaptive immune functions, including the regulation of T cells [43], it is reasonable to extrapolate that they may also negatively impact modern T cell-based immunotherapies. For example, MDSCs and TAMs are known to create an immunosuppressive TME that hinders effective T cell responses. Their suppressive repertoire includes direct inhibition of T cell cytotoxic activity and promotion of immune evasion by depletion of essential nutrients such as arginine and cysteine, which are critical for T cell receptor (TCR) ζ chain synthesis [359, 360] or abundant release of ROS, to which tumor-reactive T cells are particularly susceptible [361]. They can also interfere with lymph node homing by downregulating L-selectin on naive T cells through the action of ADAM-17, a disintegrin and metalloprotease that cleaves L-selectin, thereby impairing T cell activation [362]. In addition, they also indirectly shift the balance between tumor reactivity and tumor tolerance by recruiting through e.g. CCL3, CCL4, and CCL5 as well as be promoting the induction of T cell-suppressive T_regs_ through the release of IL-10 or TGF-β [363], among others.

MDSCs significantly influence resistance to ICIs, thereby impacting treatment outcomes in several cancer types [364]. Their circulating levels often correlate with poor prognosis and reduced response to ICI, making them critical players in immune resistance mechanisms. Several studies have shown that patients with lower baseline levels of MDSCs in their peripheral blood respond better to ICIs with nivolumab and ipilimumab. For example, in patients with non-small cell lung cancer (NSCLC), nivolumab responders had significantly fewer M-MDSCs [365]. Similarly, in melanoma, lower initial M-MDSC baseline levels were associated with improved survival and more effective immune responses towards ipilimumab and nivolumab [291, 366]. In fact, the role of MDSCs goes beyond baseline levels. Changes in MDSC populations during treatment have also been shown to influence clinical outcomes. For example, in melanoma patients treated with ICIs, those who had an early reduction in MDSCs after treatment initiation had longer overall survival and better therapeutic responses [367]. Hence, this reduction of MDSC levels appears to allow for better expansion and activation of CD8 + T cells, which are critical for the anti-tumor effects of ICIs. The inverse relationship between M-MDSC numbers and CD8^+^ T cell responses highlights the importance of MDSCs in shaping the effective immune response to ICI [368]. In addition, genes and chemokines associated with recruitment and activation of MDSCs such as CXCL2, CCL23 and IL-1β in the TME are associated with poor response to ICIs in e.g., melanoma or NSCLC [369, 370]. In a seminal study, Jiang et al., demonstrated that MDSCs signatures could predict patients’ response towards anti-PD-1 and anti-CTLA-4 treatment [371].

Similar to MDSCs, TAMs represent a heterogeneous population of immune cells within the TME that play a critical role in resistance to ICI therapies. TAMs exhibit significant phenotypic and functional diversity, which complicates their role in cancer and their impact on ICI, since different subsets of TAMs can either promote immune suppression and tumor progression or, in some cases, aid in anti-tumor immunity [372]. In many solid tumors, such as renal cell carcinoma (RCC) [373] and triple-negative breast cancer (TNBC) [374], a predominance of M2-like TAMs has been associated with poor responses to ICI therapies. TAMs influence ICI efficacy through multiple mechanisms, including the expression of immune checkpoint ligands such as PD-L1, which is the actual target of PD-1/PD-L1 blockade [375]. In certain cancers, such as nasopharyngeal carcinoma (NPC), the density of TAMs correlates with higher PD-L1 expression on tumor cells [376], suggesting that targeting PD-L1 on TAMs (and not necessarily on tumor cells) may facilitate therapeutic response to checkpoint inhibitors. This clinical observation is supported by data from preclinical models suggesting that the relevant target for ICI is PD-L1 on myeloid and non-malignant cells [377]. Furthermore, the tissue context in which TAMs are found is critical in determining their impact on ICI resistance, based on studies showing that TAMs in liver tumors, but not in lung metastases, induced macrophage-mediated resistance to ICI [378]. In addition, TAMs can reduce the efficacy of ICSs by capturing anti-PD-1 antibodies through Fcγ receptor (FcγR) binding, leading to therapeutic resistance. This Fc-FcγR interaction, observed in both human and mouse models, impairs the efficacy of ICI and may even induce hyperprogression in some NSCLC patients [379]. Though the exact mechanism behind this remains unclear, it highlights the necessity to further study the potential of the inhibition of FcγR expression on macrophages to improve ICI outcomes and avoid adverse effects such as resistance and tumor hyperprogression [380]. Consequently, targeting TAMs could harness the therapeutic efficacy of ICI [381], which is currently being explored in various clinical trials [382].

In addition to the role of MDSCs and TAMs, neutrophils have also emerged as critical players in the modulation of immune responses during ICI therapy. The neutrophil-to-lymphocyte ratio (NLR), derived from the absolute neutrophil count (ANC) and absolute lymphocyte count (ALC) in peripheral blood, serves as a reliable predictive biomarker of ICI efficacy. NLR reflects the balance between pro-tumor inflammation, driven by neutrophils, and anti-tumor immunity, mediated by lymphocytes. Higher NLR levels are often associated with poor prognosis, while lower levels indicate better therapeutic outcomes in cancers such as melanoma [383] and NSCLC [384] treated with ICIs. In addition, the dynamics of NLR after the start of ICI-based therapy can predict outcome, so that an increase in gastric cancer [385] is a negative predictor, while a decrease in RCC [386] and NSCLC [386] is a positive predictor. In summary, the NLR provides valuable insights into the balance of immune regulation in cancers as a non-invasive and effective biomarker to guide ICI therapy across multiple cancer types.

Neutrophils contribute to the induction of immunosuppression and intensify tumor vascularization, thus playing a role in the development of resistance to existing treatment options. Recent studies confirmed the synergistic effect of neutrophil depletion or modulation of their activity with other treatment modalities helps to overcome the resistance to the therapy. SB225002, a selective inhibitor of CXCR2, significantly reduces infiltration of neutrophils and enhances anti-tumor T cell activity, promoting the therapeutic effect of cisplatin [387]. Depletion of neutrophils enhanced the efficacy of checkpoint inhibition therapy [251, 388]. Also co-depletion of CXCR2^+^ tumor-associated neutrophils overcomes the compensatory response of neutrophil mobilization by depletion of CCR2^+^ tumor-associated macrophages [389]. Synergistic effect of neutrophil repolarization with other treatment modalities helps to overcome the resistance to the therapy. Targeted inhibition of fatty acid transport protein 2-dependent PGE2 synthesis by selective pharmacological inhibitor lipofermata reversed immunosuppressive activity of neutrophils, and improves the efficiency of anti-CTLA4 immune checkpoint inhibitor [390].

On the other hand, immune-activating neutrophils are reported to augment the efficacy of cancer immunotherapies, including checkpoint inhibitors and ADCC-based therapies. The capacity of neutrophils to modulate the TME and enhance immunotherapy outcomes is exemplified by their anti-tumor subtypes, which promotes a more immunogenic TME, thus improving the efficacy of immune checkpoint inhibitors [391]. Studies have shown that neutrophils are indispensable for complete tumor eradication following T cell-based immunotherapies, as evidenced by their essential role in both mouse models and biopsies from melanoma patients treated with checkpoint blockade [391].

CAR T cell therapy has emerged as the most successful adoptive cell therapy to date and is currently approved for multiple indications in hematologic B cell malignancies. Although CAR T cells are engineered to act autonomously and are cultured ex vivo in the presence of stimulatory molecules, their efficacy is still compromised by the TME. Interestingly, the immunosuppressive influence of myeloid cells begins prior to the generation of CAR T cells, starting with the leukapheresis product used to generate autologous CAR T cells [392]. In diseases such as diffuse large B cell lymphoma (DLBCL), for which CAR T cell therapy is approved, the increased frequency of circulating MDSCs have been reported [393]. These MDSCs can accumulate in the leukapheresis product and negatively affect the quality of the CAR T cell product through their T cell suppressive activity. Studies in lymphomas treated with CD19-specific CAR T cells have shown that both circulating MDSCs and immunosuppressive myeloid signatures within the TME are crucial factors in determining treatment response [394–396]. In contrast to hematological malignancies, the success of CAR T cell therapy in solid tumors has been less impressive, largely due to the challenging TME in solid tumors, which is more difficult to overcome [397]. Myeloid cells play a pivotal role in this context. For instance, a study with GD2-specific CAR T cells demonstrated that an increase in circulating PMN-MDSCs was associated with poorer treatment outcomes in neuroblastoma [398]. The interaction between myeloid cells and CAR T cells not only hampers the function of CAR T cells but also contributes significantly to the development of two major CAR T cell-associated side effects: cytokine release syndrome (CRS) and immune effector cell-associated neurotoxicity syndrome (ICANS) [399]. Activated CAR T cells trigger myeloid cells to release large quantities of pro-inflammatory cytokines, which are key contributors to these pathologies. There are preventive strategies aimed at mitigating this cytokine storm, such as the use of IL-6 or GM-CSF blocking antibodies, which may also enhance the efficacy of CAR T cells by targeting the myeloid components of the TME [400–402]. A more direct approach involves newer generations of CAR T cells designed to release cytokines such as IL-15 or IL-18, which can repolarize myeloid cells at the tumor site, enhancing the anti-tumor efficacy of CAR T cells [403, 404]. These advancements represent promising strategies to not only counteract the immunosuppressive TME but also improve CAR T cell functions in solid tumors and beyond.

Besides interfering with T cell based immunotherapies, myeloid cells were demonstrated to decrease sensitivity to other therapeutic modalities. For instance, NETs can influence chemotherapy efficacy by either enhancing or reducing drug penetration, depending on the context [405]. In some cases, NETs have been shown to hinder the diffusion of doxorubicin, reducing its ability to induce apoptosis in ovarian cancer cells [406]. Neutrophils can also interfere with anti-angiogenic therapies. For example, the infiltration of CD11b^+^ Gr1^+^ myeloid cells (neutrophils) increased tumor growth and vascularization by producing MMP9 in Balb/c mice bearing MC colorectal cancer cells [407].

### Turning myeloid cells against tumors through genetic engineering

**Fig. 2:**
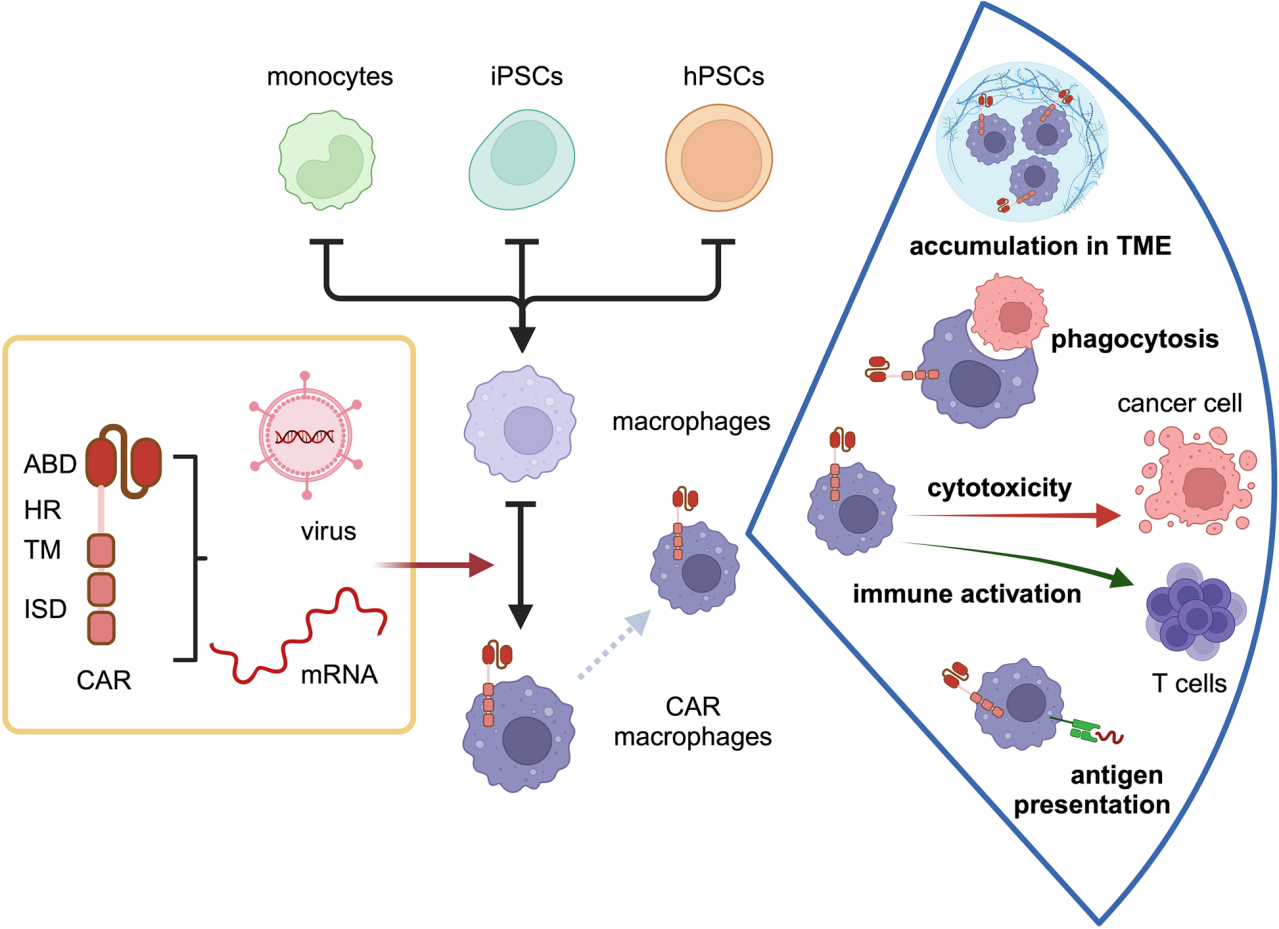
CAR macrophage (M)-based therapy in cancer. Macrophages are derived from primary monocytes, induced pluripotent stem cells (iPSCs) or hematopoietic pluripotent stem cells (hPSCs). After differentiation into macrophages, genetic modification is usually performed by viral gene transfer of DNA or mRNA-based to achieve expression of the chimeric antigen receptor (CAR). Classically, the CAR consists of an antigen-binding domain (ABD), a hinge region (HR), a transmembrane domain (TM) and an intracellular signaling domain (ISD). The resulting CAR M has several beneficial properties: they accumulate in the tumor microenvironment, they can phagocytose tumor cells, they release cytotoxic molecules and anti-tumor cytokines that directly destroy tumor cells, they activate T cells via their pro-inflammatory cytokines, and they can also present antigens

In addition to T cells, alternative cell types are being explored as “CAR-carriers”, including macrophages. Macrophages have a natural ability to accumulate and persist in the TME, making them attractive candidates for adoptive cell therapy in cancer. Their intrinsic anti-tumor functions beyond accumulation in close proximity to their target cells, such as (antigen-dependent) phagocytosis, release of cytolytic molecules such as LL-37 and ROS, and antigen presentation properties further enhance their therapeutic potential [408]. In addition, macrophages can secrete MMPs that degrade the ECM of the TME, a significant physical barrier to CAR T cell infiltration [409]. Preclinical models have shown that CAR macrophages (CAR M cells) efficiently accumulate within the TME. A potential limitation of primary CAR M cells as compared to CAR T cells is their limited ability to expand *in viv*o and their reduced persistence. However, this may also be beneficial from a safety standpoint, as temporary activation and presence may reduce the risk of difficult-to-control toxicities, particularly those associated with CRS and ICANS [399]. CAR M cells have also been observed to exert "bystander effects" by secreting pro-inflammatory cytokines that activate other immune cells, such as T cells, to target tumor cells [410], reduce the efficacy of anti-VEGF antibody therapy, and undermining its therapeutic potential [411, 412] (Fig. 2).

The basic design of macrophage CARs includes four major components [413]: An antigen-binding domain, usually a single-chain variable fragment (scFv) derived from murine or human antibodies, that binds to target cell antigens; a hinge domain that provides flexibility between the antigen-binding domain and the transmembrane domain, allowing the CAR to adapt to different spatial configurations of the antigen on target cells, thereby increasing its binding efficiency; a transmembrane domain (derived from molecules such as CD8 or CD147) that anchors the receptor to the cell membrane, providing stability and facilitating signal transduction by linking the extracellular antigen-binding domain to intracellular signaling domains; and a cytosolic signaling domain (such as CD3ζ, FcγR, or CD147) that promotes phagocytosis, MMP release, and cytokine production. A co-stimulatory domain further enhances its activity. In addition, due to the phenotypic plasticity of macrophages, it is critical to maintain their anti-tumor M1 polarization within the TME, with polarization stability strongly determined by CAR design. For example, a recent study has shown that combining CD3ζ with the Toll/Interleukin-1 receptor (TIR) domain of the TLR4 receptor within the intracellular signaling domain can maintain a stable anti-tumor phenotype within the TME [414]. Genetic modification of myeloid cells to express CARs can be challenging due to their high expression of foreign nucleic acid receptors [415]. Lentiviral and adenoviral vectors are commonly used for transduction, the latter facilitated by the high expression of CD46, a docking molecule for group B adenoviruses on macrophages [416]. In addition, mRNA-based strategies for transient CAR expression in macrophages are under preclinical investigation [417].

Macrophages can be derived from the patient’s autologous monocytes within peripheral blood mononuclear cells (PBMCs) or from purified CD14^+^ monocytes that can be differentiated into macrophages by cytokine treatments such as GM-CSF (before or after transduction) [416]. GM-CSF promotes M1 polarization, which enhances their anti-tumor properties [226]. The large quantities of cells required for this production strategy and their propensity for premature activation during isolation, can complicate the engineering process and translation into clinical settings. Alternative sources include human pluripotent stem cells (hPSCs) [418] or induced pluripotent stem cells (iPSCs) [414, 419], which can be derived from PBMCs of healthy donors that need to be reprogrammed back into iPSCs. Allogeneic stem cells offer advantages in scalability due to their self-renewal potential and cost effectiveness, making them suitable for "off-the-shelf" applications. In addition, the risk of GvHD is significantly lower with allogeneic CAR M cells than with CAR T cells due to their limited circulation time and in vivo expansion.

Preclinical data from models of e.g., leukemia of pancreatic cancer [420]. However, safety concerns regarding hPSC-/iPSC-based therapies must be addressed, particularly the risk of [417] malignant transformation due to the high proliferative potential in undifferentiated states. This risk is compounded by the possibility of insertional mutagenesis during viral gene transfer. Strategies to mitigate these risks include the use of the aforementioned mRNA-based CAR expression and CRISPR-Cas9-mediated gene editing, both of which are currently in preclinical evaluation. Several clinical trials are underway, as summarized in Table 1. These developments underscore the growing potential of macrophages as an alternative to T cells in CAR-based therapies, offering unique advantages, especially in solid tumors. However, the optimal design of CAR M cells and the mitigation of associated risks, such as ensuring their persistence, safety, and anti-tumor efficacy still remain critical areas for ongoing research and clinical evaluation.

Neutrophils genetically modified to express CARs represent a cutting-edge approach. CAR-neutrophils, engineered from progenitor cells or pluripotent stem cells [421], have demonstrated superior tumor-targeting abilities and cytotoxicity against tumor cells [421, 422], particularly in glioblastoma models, where they efficiently cross the blood–brain barrier and reach intracranial tumors [423]. This approach highlights the potential of CAR-neutrophils in treating solid tumors, where their inherent migratory and cytotoxic properties can be harnessed for precise, targeted therapy.

Adoptive transfer of reactivated neutrophils, engineered to reduce their immunosuppressive functions or enhance their cytotoxicity, represents an innovative therapeutic approach. Neutrophils deficient in pro-tumorigenic factors, such as Smad3 [262] or Tollip [424], have been shown to suppress tumor growth when transferred to tumor-bearing mice. Transfusion of IRAK-M^−/−^ neutrophils rendered an enhanced anti-tumor immune response in the murine inflammation-induced colorectal cancer model [425]. Adoptive transfer of neutrophils with inhibited NAMPT [258] or SIRT1 [142] decreased tumor angiogenesis and growth in murine models. These reprogrammed neutrophils not only directly target tumors but also modulate the TME to promote a more robust anti-tumor immune response.

While certain therapeutic agents alone have the limited capacity to reach the tumor site, and are distributed systemically, neutrophils can act as living drug delivery systems, transporting therapeutic agents specifically to the tumor tissue [426, 427]. Conjugation of liposomal stimulator of IFN genes (STING) agonists on the surface of neutrophils allows the targeted delivery of them to the tumor, potentiating the local anti-tumor immunity [428]. In preclinical models, neutrophils have been used to deliver nanoparticles (NPs) loaded with chemotherapeutic drugs, significantly improving drug delivery to tumors while reducing systemic toxicity [429]. For example, paclitaxel nanocrystals coated with an anti-CD11b antibody can target activated neutrophils, which transport the drug across the tumor vasculature, enhancing drug accumulation within the tumor [430]. Similarly, neutrophils have been employed to carry PEGylated liposomal doxorubicin for residual tumor treatment following high-intensity focused ultrasound ablation [431]. Neutrophils' ability to cross barriers such as the blood–brain barrier and bone marrow-blood barrier positions them as ideal carriers for treating metastases, especially in challenging locations like the brain and bones [432, 433]. Additional enhancement of uptake of NPs by circulating neutrophils using modification with sialic acids [434–436] or bacterial fragments [437] significantly improves their therapeutic potential. A combined approach includes the use of genetically engineered CAR-neutrophils to deliver therapeutic agents, such as hypoxia-targeting tirapazamine-loaded silica NPs [423], activated by hypoxic tumor microenvironment.

Neutrophils are also being explored as carriers for photodynamic and photothermal therapies. These therapies utilize externally generated ROS or heat to kill tumor cells. Neutrophils loaded with photosensitizers, such as pyropheophorbide-a-loaded albumin NPs, have been shown to improve the efficacy of photodynamic therapy in restraining tumor progression and extending lifespan in murine models [426]. Similarly, gold nanorods taken up by neutrophils have demonstrated enhanced tumor-targeting ability for photothermal therapy [438], offering a promising avenue for tumor-specific thermal ablation and combinational therapy [439]. “Photoactive neutrophils” were reported to carry photosensitizer Ce6 directly to cancer cell, thus elevating their cytolysis [440, 441]. Loading of neutrophils with NPs of particular types, detectable by various imaging modalities (MRI, ultrasound etc.), allows to combine tumor imaging with therapy [442–445].

Another innovative approach is using neutrophil membranes alone. Neutrophil membrane fragments inherit the surface proteins and targeting properties of neutrophils, which allow them to evade immune detection, home to tumor sites and carry there their cargo (NPs, chemotherapeutic agents), protecting it from the degradation in the bloodstream [446–448]. Neutrophil membrane-camouflaged NPs have been employed to deliver drugs directly not only to the tumor cite, but also to circulating tumor cells (CTCs) [449–451], thus successfully eradicating them and reducing metastatic spread, with a potential to use in CTC isolation and diagnostics. Loading of neutrophil membrane with a set of neutrophilic enzymes for ROS production (so called "synthetic neutrophils”) [452, 453] opens the possibility of specific utilization only of a single “neutrophil” subset with specific functionality without involving neutrophils with other properties. Moreover, neutrophil membranes could potentially serve as a scavenger of tumor-produced immunosuppressive cytokines and chemoattractants, thus limiting the expansion of immunosuppressive cell populations within tumor [454].

In addition to using neutrophils directly for drug delivery, researchers are investigating the potential of neutrophil EVs for therapeutic purposes. EVs, such as exosomes, are nanosized vesicles secreted by cells, including neutrophils, and can serve as natural carriers of therapeutic agents. Neutrophil-derived exosomes have shown potential for delivering chemotherapeutic agents like doxorubicin in tumor models [455, 456]. These exosomes inherit the targeting properties of neutrophils, allowing them to home to tumor sites and deliver drugs effectively, while their biocompatibility and low immunogenicity make them an appealing platform for drug delivery.

### Discussion and future perspectives

It is well documented that myeloid cell-based treatments offer a diverse array of therapeutic options, from direct cytotoxic functions to drug delivery and cellular therapy. By targeting myeloid cells' unique properties, these therapies provide new avenues for improving cancer treatment outcomes, particularly in solid tumors. With further refinement, myeloid-targeted strategies could become a valuable addition to the current cancer therapy landscape, offering both precision and efficacy while minimizing adverse effects.

As part of myeloid cell family, macrophages possess the ability to engulf tumor cells and present tumor-specific antigens, thus contributing to adaptive anti-tumor immunity, while these also facilitate immune escape of tumor cells through cytokines such as CD47 secreted by the tumors [457]. Consequently, strategies targeting macrophage phagocytosis by blocking anti-phagocytic signals and tumor efferocytosis show promise for cancer immunotherapy. Furthermore, the phenotype repolarization of TAMs is considered another strategy for efficient cancer therapy, since most TAMs are polarized into pro-tumor M2 phenotype with dynamic and heterogeneous property by the cytokine stimuli in the TME [210]. To this end, studies aimed at identifying anti-phagocytic signals or cytokines/receptors of myeloid cells in the TME as a therapeutic target for efficient cancer immunotherapy have been studied. Recently, Jing et al*.* suggested that strategies to harness myeloid cells include inhibition of myeloid cell production, recruitment, direct depletion, regulation of cell surface receptors and reprogramming of myeloid cells [212]. However, despite numerous clinical and translational studies performed over the years using antibodies and small molecule inhibitors targeting CCL2/CCR2, PI3Kγ, CXCL2/CXCL8, CD47/SIRPα, CSF1R, TLR7/8 and LOX1 as myeloid modulators, the outcomes have largely been modest, with limited efficacy and resistance to immunotherapy. A notable exception is pexidartinib, a CSF-1R inhibitor approved by the FDA for tenosynovial giant cell tumor [458]. Also, several promising myeloid cell modulators include CXCR2 inhibitor AZD5069, the PI3Kγ inhibitor eganelisib, the anti-IL-8 antibody BMS-986253, the CCR2/5 antagonist BMS-813160 and the CD40 agonist antibody selicrelumab, which enhances T cell response. However, for effective treatment it should be considered that the efficacy of anticancer agents can vary depending on the tumor types, as Casanova-Acebes et al*.* [312] suggest that CCR2 inhibitors are not effective in CCR2 mutant NSCLCs due to the compensation by TAMs or M-MDSCs.

The emerging field of neutrophil-targeted therapies in cancer presents both promising opportunities and significant challenges. Neutrophil diversity within the TME, characterized by the presence of both pro-tumorigenic and anti-tumorigenic neutrophil populations, necessitates a careful approach when targeting neutrophils therapeutically. Unspecific depletion of neutrophils can inadvertently remove populations that may contribute to tumor elimination. In certain contexts, neutrophil depletion has been linked to increased tumor burden [459], indicating their potential role in anti-tumor immunity. For instance, granulocytes, when isolated from healthy animals and administered to the tumor site in experimental models have been shown to reduce tumor growth and enhance survival rates in tumor-bearing animals [460]. Similarly, mature granulocytes when introduced intratumorally have demonstrated anti-tumor effects, contrasting with pro-tumor roles of PMN-MDSCs [461].

Systemic drug administration influences various cell types, thus being responsible for side effects. To address this, neutrophil-specific approaches have been developed to minimize the undesirable systemic adverse effects. Cellular therapy strategies show promise in enhancing neutrophils' anti-tumor activity. Furthermore, neutrophils' strong chemotactic properties make them ideal vehicle for targeted drug delivery and therapies that rely on their ability to home to tumor sites. For the further enhancement of specificity of neutrophil targeting, identifying and targeting unique surface markers or their combinations specific to tumor-promoting neutrophil subsets represents an essential goal. The use of bi-specific antibodies (BsAbs) offers a promising avenue for targeting unique surface markers or metabolic pathways specific to tumor-promoting neutrophil subsets. BsAbs are engineered to simultaneously bind two or even three different antigens, enabling precise targeting of distinct cellular populations or pathways within the TME [462]. High specificity of BsAbs is achieved by targeting simultaneously two target antigens on different cell types (myeloid cells, lymphocytes, tumor cells), reducing off-target effects. By engaging multiple pathways or cell types, BsAbs amplify therapeutic effects, such as combining immune activation with tumor cell killing. Moreover, BsAbs can be tailored for various combinations of targets, making them adaptable to different cancer types and TME conditions. Overall, bi-specific antibodies represent a cutting-edge approach to harnessing the complexity of neutrophil biology in cancer therapy, offering new opportunities for precision medicine in oncology.

However, approaches targeting neutrophils could be associated with potential side effects and challenges. One major concern is the risk of compromising systemic immunity. Neutrophils play a crucial role in defending against pathogens, and their depletion or modulation could potentially increase susceptibility to infections [463]. This is particularly concerning in cancer patients who may already have compromised immune systems [464]. On another hand, elevated tissue toxicity of neutrophils might also lead to compromised immune responses against certain pathogens [465], and also lead to autoimmune reactions [466]. Another potential side effect is the disruption of normal wound healing processes, in which neutrophils play a key role [467]. This could lead to delayed recovery from surgical interventions or other tissue injuries. The potential side effects affecting lymphocytes, would likely depend on the specific targeting strategy used and the extent of neutrophil depletion or modulation, varying from reduced activation of adaptive immunity in case of neutrophil depletion to exaggerated lymphocyte cytotoxicity. Careful monitoring of immunological responses would be necessary in clinical applications of neutrophil-targeted therapies.

Current literature highlights the critical role of eosinophils within TME, showcasing both pro- and anti-tumorigenic functions influenced by tumor type, the stage and the surrounding TME. Therefore, these variables should be taken into account for rationally based interventions that either enhance or inhibit eosinophils activity. Special attention should be posed to the heterogeneity and activation state of eosinophils. Current studies suggest that some signals, such as IFN-γ and IL-33, promote intratumoral accumulation of eosinophils and polarize them towards anti-tumor functions. Eosinophils have also demonstrated significant contributions to ICB therapies, suggesting their potential to extend these therapies’ efficacy to a wider range of patient population. Additionally, given the strict interactions with T cells in the TME, eosinophils represent promising candidates to support CAR T cells facilitating their entry into the tumor through release of T cell-attracting chemokines (i.e., CXCL9, CXCL10) [468]. In this regard, the combination of human pluripotent stem cell-derived eosinophils with CAR T cells exhibited synergistic anti-tumor effects in preclinical models [469], underscoring the potential use of eosinophils to augment the efficacy of CAR T cells. On the other hand, since eosinophils release also Th2-skewing cytokines and pro-angiogenic factors, a prolonged persistence of these cells in the TME may in the end favor tumor progression. This aspect as well as the tumor location must be taken into account in view of future eosinophil-based therapeutic strategies. In this respect, the development of locally delivered EV-based therapies, including engineered immune cell-derived EVs combined with anti-cancer drugs, represents another promising therapeutic avenue to timely deliver anti-tumorigenic molecules circumventing unwanted reactions.

While CAR T cell therapy has demonstrated a promising efficacy in treating hematologic malignancies, its application remains limited in solid tumors due to the several harmful adverse effects [470]. In contrast, CAR M cells offer a potential solution for solid tumors. These cells can effectively reprogram M2-type macrophages into an M1-type macrophages and recruit T cells to fight against the growth of solid cancers [14].

Additionally, combination therapies with immune checkpoint inhibitors or other forms of immunotherapy are recommended to overcome the limitations and adverse events associated with myeloid cell modulators in several clinical trials. Although several SIRPα-CD47 inhibitors have shown a good efficacy in hematologic cancers, combination therapies with agents such as cetuximab, rituximab, azacitdine, avelumab are recommended, as the antitumor activity of SIRPα-CD47 inhibitors is less pronounced in solid tumors.

Emerging evidence underscores the role of dendritic cells (DCs) as antigen-presenting cells critical for initiating T cell responses [471]. Lapenta et al*.* [472] highlighted that a personalized vaccine based on dendritic cells generated in the presence of IFN-α and GM-CSF (IFN-DC) loaded with (HOCl)-oxidized MCF-7 cell lysate impeded the growth of MCF-7 cells in NOD SCID mouse model. Furthermore, Phase II clinical trial (NCT03400917) revealed that AV-GBM-1 (a tumor-initiating cell targeted dendritic cell vaccine) increased median progression-free survival (mPFS) compared to untreated control group.

Nonetheless, inflammatory toxicity by checkpoint blockade and CAR T cell therapy is also a major concern as a major side effect to disturb the efficacy of myeloid cell targeted treatment and their combination therapy [473]. Emerging evidence reveals that recruitment and activation of myeloid cells by T cells enhance local and/or systemic release of proinflammatory cytokines such as IL-1β, IL-6 and TNF-α that play a critical in these inflammatory toxicities [474] and also cytokine release syndrome induced by CAR T cells [475]. Thus, further studies are required to define the cellular and molecular signaling at nontoxic concentrations or doses to prevent inflammatory toxicities and sustain anti-tumor immunity.

In summary, to improve the success of myeloid cell-targeted therapy by repolarizing macrophages into M1 like phagocytes some critical steps are required. First, it is essential to determine tumor types and patient subsets through profiling to identify the dominant drivers of therapy resistance in tumor-associated myeloid cells. Additionally, selecting the most efficacious treatment regimens and combination drugs is crucial to enhance synergy and to minimize side effects. Moreover, the sensitivity of myeloid cell modulators such as antibodies or small molecule inhibitors or dendritic cell vaccine should be rigorously evaluated in blood cancers or solid tumors. This evaluation should be conducted in vitro, in vivo or in patient derived xenograft (PDX) models before initiating translational and clinical studies. Also, the choice of therapies such as CAR T cell therapy, CAR M therapy, or SIRPα-CD47 inhibitor should be determined based on tumor types such as solid tumors or hematologic malignancy.

From a clinical perspective, further comprehensive studies are needed to evaluate the long-term efficacy and safety of the neutrophil targeted therapies across various cancer types. The development of reliable biomarkers to predict and monitor response to myeloid-targeted therapies will be crucial. This could involve measuring neutrophil-to-lymphocyte ratios, assessing myeloid cells phenotypes in blood or tumor samples, or monitoring levels of neutrophil-derived factors like NETs or specific cytokines.

**Fig. 3:**
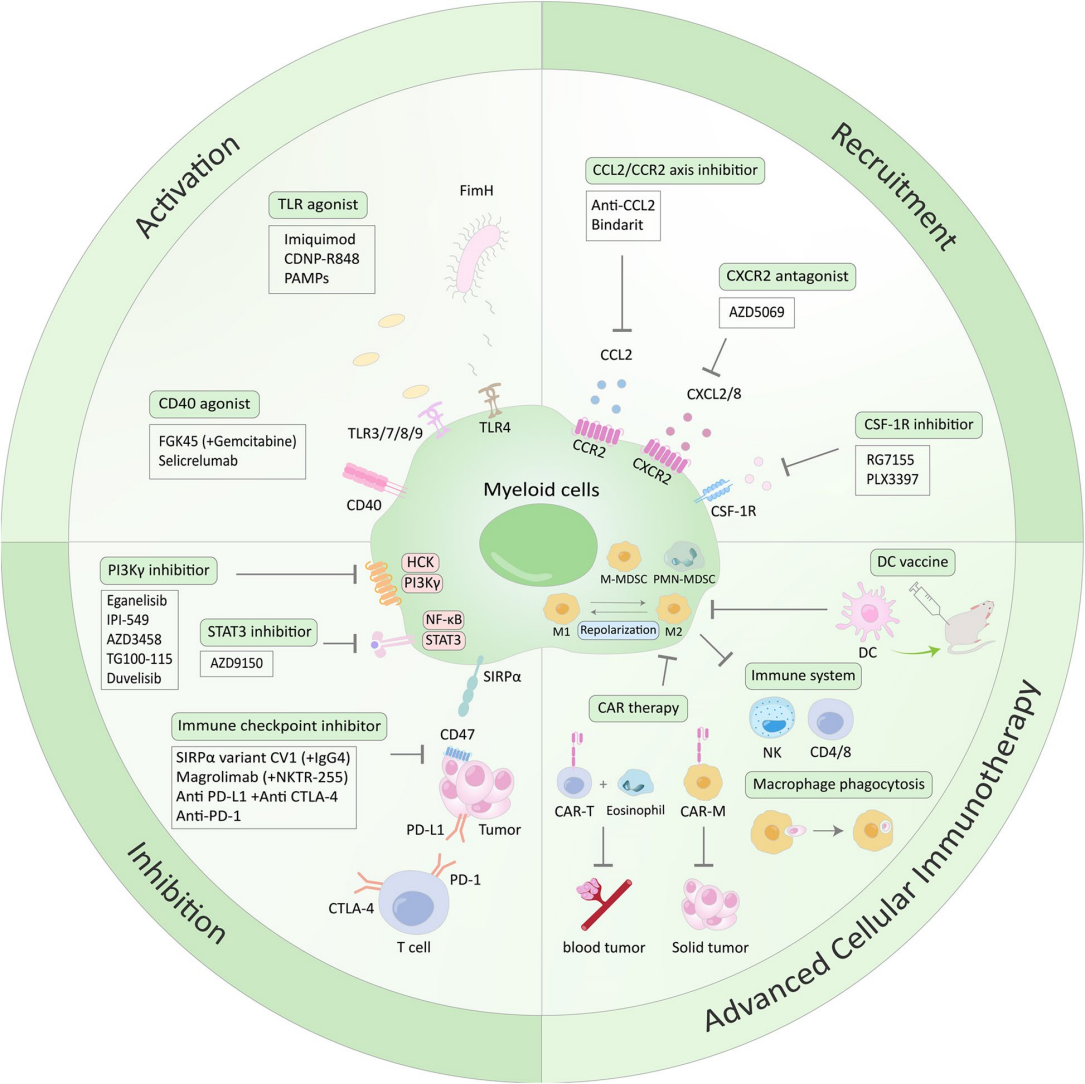
Therapeutic targets, modulators and advanced cellular therapies of myeloid cells in the tumor microenvironment. (i) Inhibitors of PI3Kγ, HCK, STAT3/NFkB, SIRPα and CD47 have been used in several cancers to repolarize M2 like macrophages into M1 like macrophages, leading to anti-tumor activity in myeloid cells. (ii) Agonists of CD40, FimH, TLR (3,7,8,9) repolarize M2 like macrophages into M1 like macrophages in myeloid cells. (iii) Inhibitors of CCL2/CCR2 or CSF1R block their recruitment on myeloid cells to exert anti-tumor activity. (iv) For advanced cellular therapy, dendritic cell vaccine can be applied to cancer patients alone or in combination with ICB. Also, CAR-T therapy can be effectively used for blood cancers, which can be promoted by eosinophils, though the safety study should be assessed in advance. Notably, CAR-M therapy can be more effectively used in solid tumors to overcome the weakness of CAR T therapy

In conclusion, while myeloid cell-targeted therapies hold great promise for cancer treatment, their development and implementation will require careful consideration of potential side effects and the complex biology of myeloid cells in the tumor microenvironment. Future research should focus on enhancing specificity, exploring combination therapies, and developing robust biomarkers to guide treatment decisions. To improve the efficacy of myeloid cell modulators, combination therapy is strongly recommended only with the robust data from in vitro, in vivo experiments and preclinical studies to ensure better outcomes in the future clinical applications (Fig. 3).

## Data Availability

No datasets were generated or analysed during the current study.
